# Metabolic responses to benzoic acid stress and glutamine transport-dependent vulnerabilities in *Escherichia coli* revealed by NMR metabolomics

**DOI:** 10.1007/s11274-026-04971-5

**Published:** 2026-04-24

**Authors:** Hatice Öztürkel-Kabakaş, Kadriye Aslıhan Onat-Taşdelen, Şükrü Serter Çatav, Eralp Doğu, Ecem Yüksektepe, Young Kee Chae, Bekir Çöl, Emine Sonay Elgin

**Affiliations:** 1 Graduate School of Natural and Applied Sciences, Biology Program, Muğla Sıtkı Koçman University, Muğla, Türkiye; 2 Department of Biology, College of Sciences, Muğla Sıtkı Koçman University, Muğla, Türkiye; 3 Department of Statistics, College of Sciences, Muğla Sıtkı Koçman University, Muğla, Türkiye; 4 Vocational School of Health Services, Pathology Laboratory Techniques Program, Fenerbahçe University, İstanbul, Türkiye; 5 Department of Chemistry, College of Natural Sciences, Sejong University, Seoul, South Korea; 6 Biotechnology Research Center, Muğla Sıtkı Koçman University, Muğla, Türkiye; 7 Center for Research Laboratories, Muğla Sıtkı Koçman University, Muğla, Türkiye; 8 Department of Chemistry, College of Sciences, Muğla Sıtkı Koçman University, Muğla, Türkiye

**Keywords:** *Escherichia coli*, Benzoic acid, Glutamine transport, NMR metabolomics, Antibacterial activity, Acid stress

## Abstract

Benzoic acid (BA) is a widely used weak organic acid preservative with antimicrobial activity, yet the metabolic basis of its antibacterial action and the determinants of bacterial sensitivity remain incompletely understood. Here we combined growth assays with ^1^H NMR metabolomics to characterize BA-induced metabolic responses in *Escherichia coli* BW25113 and to examine metabolic changes associated with impaired glutamine transport. Wild-type BW25113 and its BA-sensitive isogenic Δ*glnP* mutant, lacking the membrane-bound glutamine permease of the high-affinity GlnHPQ transport system, were exposed to sublethal BA concentrations. BA slowed growth and significantly altered the levels of 42 metabolites in the wild-type and 38 in Δ*glnP*, with the mutant showing stronger growth inhibition and reduced BA tolerance. Both strains exhibited metabolic changes consistent with cellular responses to oxidative and acid stress, including alterations in central carbon metabolism, lysine degradation, cysteine and methionine metabolism, pyrimidine metabolism, and one-carbon pool by folate. However, several metabolic responses differed between the two strains. In wild-type cells, BA exposure was associated with changes in glycerolipid metabolism, glycerophospholipid metabolism, nicotinate and nicotinamide metabolism, lysine biosynthesis, glycine, serine and threonine metabolism, and purine metabolism. In contrast, Δ*glnP *cells showed distinct alterations in D-amino acid metabolism, arginine biosynthesis, and other carbon fixation pathways. In addition, the mutant displayed substantial baseline differences relative to the wild-type, including altered nucleotide and amino acid pools. Together, these results indicate that both BA exposure and deletion of *glnP* induce broad metabolic adjustments in *Escherichia coli*. Loss of *glnP* is associated with distinct metabolic states and altered responses to BA stress, highlighting the importance of glutamine transport in adaptation to weak organic acid stress.

**Supplementary information:**

The online version contains supplementary material available at 10.1007/s11274-026-04971-5.

## Introduction

Benzoic acid (BA), a phenylcarboxylic acid naturally present in various fruits and fermented products, is widely used as antimicrobial preservative in food, cosmetic, and pharmaceutical formulations (Sieber et al. 1995; Brul and Coote 1999; del Olmo et al. 2017; Issa and Mohammed 2025). Its antibacterial activity has long been recognized across diverse bacterial species (Bosund 1963; Chipley 2005). The primary mode of action is thought to involve disruption of intracellular pH homeostasis: in its protonated form, BA diffuses across the bacterial membrane and releases a proton in the cytoplasm, resulting in acidification (Salmond et al. 1984; Praphailong and Fleet 1997; Pernin et al. 2019). BA and its derivatives have also been reported to impair membrane function (Hazan et al. 2004), interfere with energy metabolism (Warth 1991a, b), and inhibit several enzymes central to cellular function, including those involved in pantothenate and coenzyme Q biosynthesis (Hilton 1965; Nishida et al. 2020) and D-amino acid oxidation (Bartlett 1948). These observations suggest that BA acts through multiple cellular pathways.

In *Escherichia coli* (*E. coli*), BA toxicity has likewise been linked primarily to intracellular acidification (Salmond et al. 1984). Additional mechanisms, such as inhibition of amino acid uptake (Eklund 1980), uncoupling of oxidative phosphorylation from the electron transport chain (Freese et al. 1973; Sheu et al. 1975), and inhibition of specific enzymes (Kruk and Lee 1982), have also been proposed. Comparative proteomic analyses of *E. coli* grown with or without BA at different pH values (6.5 vs. 8.0) further suggest that coping mechanisms extend beyond acid stress responses (Lambert et al. 1997). Despite its longstanding use, however, the broader metabolic consequences of BA stress and the genetic determinants that modulate bacterial sensitivity remain incompletely understood.

The *E. coli* KEIO single-gene knockout collection (Baba et al. 2006) provides a powerful resource for identifying genetic factors that influence antimicrobial responses. Our previous work using propolis water extract, a complex antimicrobial mixture, identified several KEIO mutants with increased sensitivity, including Δ*glnP* (Dibek et al. 2025). Notably, Δ*glnP* also displayed increased sensitivity to BA–a phenolic acid commonly encountered in propolis at biologically relevant concentrations (Elgin et al. 2023)–compared with the parental BW25113 strain (preliminary data). The *glnP* gene encodes the membrane-bound glutamine permease of the high-affinity glutamine transport system GlnHPQ in *E. coli* (Nohno et al. 1986). Glutamine is central to nitrogen metabolism, donating its amide group for the biosynthesis of amino acids, nucleotides, and amino sugars (Reitzer 2003; Miyakoshi 2024) and contributing to redox balance and energy metabolism (Forchhammer 2007; Aldarini et al. 2017). The BA-sensitive Δ*glnP* phenotype therefore suggests a possible link between glutamine transport, nitrogen metabolism, and BA tolerance. However, whether this sensitivity arises solely from the role of glutamine in cellular processes that contribute to acid resistance remains unclear. In *E. coli*, glutamine is a central nitrogen donor that supports nitrogen assimilation via glutamine synthetase (GS)-glutamate synthase (GOGAT) pathway, and the biosynthesis of several amino acids, including glutamate and arginine (Reitzer 2003). These amino acids participate in amino-acid dependent acid resistance systems, such as the glutamate- and arginine-dependent decarboxylation pathways that consume intracellular protons and help maintain cytoplasmic pH under acidic conditions (Foster 2004). Alternatively, additional mechanisms related to metabolic or regulatory effects of impaired glutamine transport may also contribute to the observed BA sensitivity. Supporting a broader connection, Hazan et al. (2004) reported that BA exposure in combination with nitrogen starvation produced a synergistic cytocidal effect in *Saccharomyces cerevisiae*, whereas either condition alone was cytostatic. Furthermore, Δ*glnP* did not display sensitivity to another phenolic acid, trans-cinnamic acid, in a genome-wide screen (Kürkçü et al. 2025), suggesting that its role in BA response may be more specific than a generalized acid-stress effect. Together, these observations reinforce the need to investigate how nitrogen metabolism intersects with BA stress responses in bacteria.

Metabolomics offers a systems-level approach to characterize cellular responses to various stresses (Johnson et al. 2016). In particular, nuclear magnetic resonance (NMR)-based metabolomics enables the simultaneous detection of diverse metabolite classes and provides a global view of metabolic perturbations under near-physiological conditions (Emwas et al. 2019; Kok et al. 2022). When combined with advanced statistical frameworks such as principal component analysis (PCA), two-way ANOVA, and ANOVA-simultaneous component analysis (ASCA), metabolomics can distinguish treatment effects, mutation effects, and their interactions (Smilde et al. 2005; Jiang et al. 2022; Pang et al. 2024). Mapping significant metabolite changes to biochemical pathways using pathway analysis tools (Xia and Wishart 2011) further enables the identification of key cellular processes perturbed by antimicrobial stress.

In this study, we combined growth assays with ^1^H NMR-based metabolomics to investigate the metabolic responses of *E. coli* BW25113 and the BA-sensitive Δ*glnP* mutant to BA exposure. Specifically, we aimed to (i) characterize the effects of BA on bacterial growth, (ii) identify global and strain-specific metabolite changes associated with BA stress and *glnP* deletion; and (iii) map these changes to metabolic pathways to better understand the physiological processes affected during BA exposure. By integrating metabolite profiling with multivariate statistical and pathway analyses, we provide a systems level view of metabolic responses to BA stress. These results offer new insight into how BA perturbs central metabolism in *E. coli* and highlight a potential link between glutamine transport, nitrogen metabolism, and bacterial tolerance to weak organic acid stress.

## Materials and methods

### Bacterial strains and chemicals

The parental *E. coli* BW25113 strain and the corresponding Δ*glnP* single-gene deletion mutant from the KEIO collection were used in this study. Benzoic acid (99% purity) was purchased from Sigma-Aldrich. A 125 mg mL^− 1^ BA stock solution was freshly prepared before each experiment. Bacteria were cultured in Luria-Bertani (LB) medium.

### Determination of sublethal BA concentrations

BA tolerance of *E. coli* BW25113 and Δ*glnP* strains was assessed using tolerance (spot) assays. Single colonies were grown overnight in LB at 37 °C with shaking (150 rpm). Kanamycin (50 µg mL⁻¹) was added for Δ*glnP* cultures. Overnight cultures were diluted in fresh LB to an OD_600_ of 0.05 and grown to mid-exponential phase (OD_600_ ≈ 0.5). Serial dilutions (1:1–1:16) were prepared in sterile phosphate-buffered saline (PBS) and 5 µL aliquots were spotted onto LB-agar plates containing BA (0.25 − 2.25 mg mL⁻¹). Plates were incubated at 37 °C for up to 48 h and photographed daily to monitor growth.

### Generation of bacterial growth curves

Growth of BW25113 and Δ*glnP* strains was monitored in 96-well microplates in the absence or presence of BA (0.25, 0.50 and 0.75 mg mL⁻¹) using a plate reader with incubation and shaking capabilities (Multiskan GO, Thermo Scientific). Five colonies of each strain were inoculated into 10 mL LB and grown overnight at 37 °C, with shaking (150 rpm). Overnight cultures were diluted in fresh LB (supplemented with kanamycin for Δ*glnP*) to an OD_600_ < 0.05, and 200 µL aliquots were dispensed into wells of sterile 96-well plates with five technical replicates per condition distributed randomly across the plate to minimize positional effects. Outer wells were filled with LB to minimize evaporation. The plate was covered with a Triton X-100/ethanol (1:9) washed lid and incubated at 37 °C with continuous shaking. OD_600_ was recorded every 30 min for 24 h.

### BA treatment of *E. coli* cells and extraction of metabolites

 For NMR metabolomics experiments, three sublethal BA concentrations (0.25, 0.50, and 0.75 mg mL^-1^) were selected based on the tolerance assays. Untreated cultures served as controls. To avoid confounding metabolic effects, kanamycin was omitted from Δ*glnP *cultures during metabolomics experiments. BW25113 and Δ*glnP *cells were grown overnight from single colonies in 100 mL LB at 37°C with shaking at 150 rpm. Overnight cultures were diluted into 200 mL LB in culture flasks (four treatment groups including control, three biological replicates each) to an initial OD_600_ of 0.05. Cells were incubated at 37°C, at 150 rpm until OD_600_ reached 0.6. BA was then added to the appropriate cultures to the indicated final concentrations and cells were incubated for an additional 1 h under the same conditions. Cultures were subsequently placed on ice and the cells were harvested by centrifugation (4000 × *g*, 15 min, 4°C). Cell pellets were washed three times with 20 mL of ice-cold PBS and stored at -80 °C until metabolite extraction. 

Metabolites were extracted using the boiled water method described by Onat-Taşdelen et al. (2024). Frozen cell pastes were suspended in 15 mL boiled ultrapure H_2_O and autoclaved (120 °C, 20 min). After cooling, samples were vortexed and centrifuged (10,000×*g*, 30 min, 4 °C). The supernatant was transferred to a previously washed (3 × 15 mL ultrapure H_2_O) 3 kDa MWCO centrifugal filters (Amicon) and centrifuged at 4 °C, (4000 × *g*) until the retained volume was < 0.5 mL. The filtrate containing metabolites was collected, frozen, freeze-dried, and quantified.

### NMR data acquisition and chenomx analysis

Freeze-dried metabolite extracts were reconstituted in 600 µL NMR buffer (10 mM sodium phosphate, pH 7.0, 0.5 mM DSS, 5% D_2_O) and transferred to 5 mm NMR tubes. Spectra were acquired at the Korea Basic Science Institute (Seoul, Korea) on a Bruker Avance II 700 MHz spectrometer equipped with a 5 mm TXI probe using a 1D NOESY (noesypr1d) pulse sequence. Spectra were collected at 298 K with a spectral width of 12 ppm and 64k data points. Water suppression was achieved by presaturation during the NOESY mixing time and relaxation delay. The NOESY mixing time, relaxation delay, and number of scans were 0.05 s, 1 s, and 32, respectively.

Spectra were processed using the Processor module of Chenomx NMR Suite (v9.0 Chenomx Inc., Edmonton, Canada). Phase and baseline corrections were applied, and spectra were referenced to the internal DSS standard (0.5 mM) following Fourier transformation and removal of the residual water signal.

Metabolite identification and quantification were performed using the Chenomx Profiler module. Automatic fitting against the Chenomx metabolite library (358 compounds) was followed by manual inspection and adjustment of assignments. On average, approximately 70% of the total spectral area was assigned with high confidence. Metabolite concentrations were scaled to the total spectral integral prior to statistical analysis. The scaling factors obtained from spectral integration were in good agreement with dry metabolite extract amounts with a few exceptions where sample loss and/or quantification errors might have occurred.

### Statistical analysis

Statistical analyses were performed using MetaboAnalyst 6.0 (Pang et al. 2024). Prior to analyses, metabolite data were normalized to constant sum, log_10_-transformed, and autoscaled. Data normality and homogeneity of variance were assessed using Shapiro-Wilk and Bartlett`s tests, respectively. One-way analysis of variance (ANOVA) and Tukey`s HSD post-hoc analysis were carried out for multi- and two-group comparisons, respectively. A false discovery rate (FDR)-adjusted *p* < 0.05 was considered statistically significant. Two-way ANOVA was performed to evaluate the effects of strain, BA treatment, and their interaction on metabolite levels.

Principal component analysis (PCA), and partial least-squares discriminant analysis (PLS-DA) were conducted separately for each strain. The quality of the PLS-DA model was evaluated by the proximity of R^2^ (goodness of fit) and Q^2^ (predictive ability) parameters to 1. Metabolites with variable importance in projection (VIP) scores > 1 were considered discriminative. ANOVA-Simultaneous Component Analysis (ASCA) (Smilde et al. 2005) was applied to evaluate the contributions of strain, BA treatment, and their interactions to the overall metabolic variation. Quantitative pathway analysis was performed to identify the pathways associated with the BA response and *glnP* deletion. The `global test` and `betweenness centrality` algorithms were applied for pathway enrichment and pathway topology analyses, respectively. The KEGG metabolic pathway database for *E. coli* K-12 MG1655 served as the reference. Statistical significance was defined as Holm-Bonferroni-adjusted *p* values < 0.05.

## Results

### Impact of BA on growth of *E. coli* BW25113 and Δ*glnP* strains

The antibacterial activity of BA on various bacteria, including *E. coli*, has long been recognized (Bosund 1963; del Olmo et al. 2017). In our previous study investigating the effects of propolis water extract (PWE) on the KEIO single-gene knockout collection, several mutants displayed increased sensitivity compared with the wild-type BW25113 strain (Dibek et al. 2025). Because BA and its derivatives occur in propolis samples from Türkiye at biologically relevant concentrations (Elgin et al. 2023), we further examined BA sensitivity in a subset of PWE-sensitive KEIO mutants (unpublished observations). These experiments indicated that the deletion of *glnP* reduces BA tolerance in *E. coli*. To gain insight into the cellular responses associated with BA stress, we therefore performed growth and metabolomics analyses of BW25113 and the Δ*glnP* mutant.

**Fig. 1:**
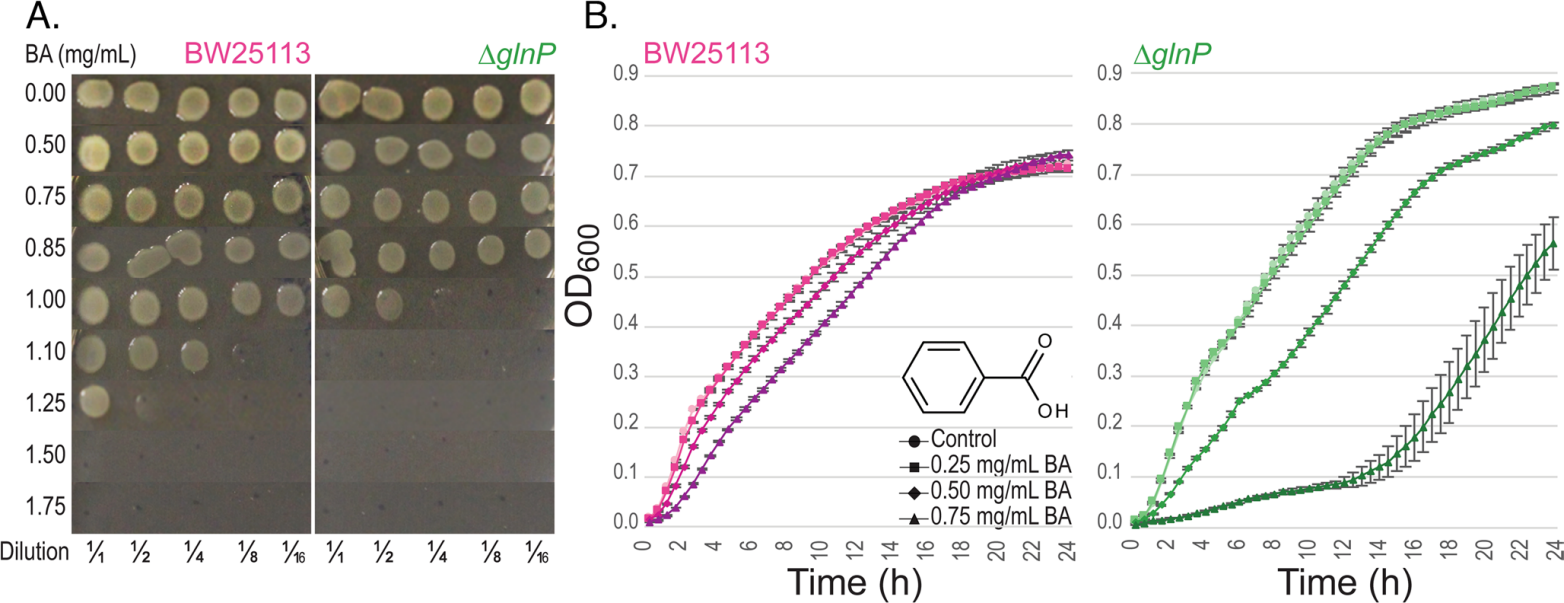
Spot assays and growth curves of *E. coli* BW25113 and Δ*glnP* strains treated with BA. (**A**) Photographs were taken after 24 h incubation at 37 °C. BA concentrations are indicated on the left (mg mL^− 1^). (**B**) Growth curves recorded in 96 well plates by measuring OD_600_ every 30 min for 24 h at 37 °C with continuous orbital shaking at medium speed

The antibacterial activity of BA toward BW25113 and Δ*glnP* strains was first assessed by spot assays. BA concentrations ≥ 1.5 mg mL⁻¹ completely inhibited growth of the wild-type strain, whereas Δ*glnP* cells displayed no detectable growth above 1.0 mg mL^−1^ (Fig. 1A), indicating increased BA sensitivity of the mutant. Based on these results, 0.25, 0.50, and 0.75 mg mL^−1^ BA were selected as sublethal concentrations for metabolomics experiments.

Growth curves were then generated to evaluate the effect of BA on growth dynamics. At 0.25 mg mL^−1^ BA, both strains showed growth patterns similar to untreated controls (Fig. 1B). At 0.5 mg mL^−1^, BA caused a clear reduction in growth rate in Δ*glnP* with approximately a two-fold delay during the first 10-h, whereas BW25113 was only mildly affected. The strongest inhibition was observed at 0.75 mg mL^−1^ BA, where Δ*glnP* exhibited markedly delayed growth reaching OD_600_ = 0.1 after ~ 12 h compared with ~ 3 h for the wild-type. Together, these results indicate that deletion of *glnP* reduces BA tolerance and impairs growth under BA stress.

### ^1^H-NMR analysis of the impact of *glnP* deletion on the metabolome of *E. coli* BW25113

**Fig. 2:**
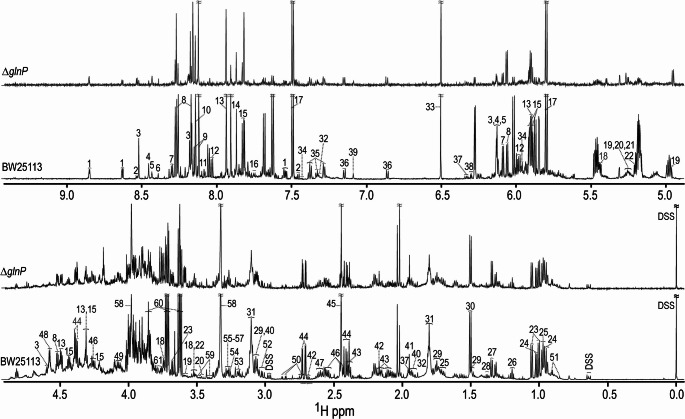
^1^H-NMR spectra of metabolites isolated from BW25113 and Δ*glnP E. coli *strains grown in LB media. Metabolite annotations are shown in the spectrum of BW25113 cells with numbers. Metabolite names corresponding to those numbers are: 1-niacinamide, 2-nicotinate, 3-AMP, 4-ATP, 5-ADP, 6-formate,7-inosine, 8-adenosine, 9-adenine, 10-hypoxanthine, 11-GTP, 12-UMP, 13-guanosine, 14-xanthine, 15-uridine, 16-cytidine, 17-uracil, 18-sucrose, 19-ribose, 20-glucose, 21-galactose, 22-trehalose, 23-valine, 24-Ileucine, 25-leucine, 26-ethanol, 27-lactate, 28-threonine, 29-cadaverine, 30-alanine,31-putrescine, 32-thymine, 33-fumarate, 34-cytosine, 35-phenylalanine, 36-tyrosine, 37-dTTP, 38-dCTP, 39-histidine, 40-lysine, 41-acetate, 42-methionine, 43-glutamate, 44-malate, 45-succinate, 46-pyroglutamate, 47-isocitrate, 48-2-phosphoglycerate, 49-serine, 50-aspartate, 51-pantothenate, 52-glutathione, 53-ethanolamine, 54-choline, 55-O-acetylcholine, 56-O-phosphocholine, 57-O-phosphoethanolamine, 58-betaine, 59-proline, 60-glycerol, and 61-fructose

In order to understand the metabolic consequences of *glnP deletion*, NMR-based metabolomics analysis was performed. Representative ^1^H-NMR spectra from the control samples of each strain, together with metabolite annotations, are shown in Fig. 2. Overall, the spectra displayed broadly similar peak patterns across strains and treatment groups relative to their respective controls (Figure S1), with differences primarily observed in signal intensities rather than peak composition. Chenomx analysis enabled identification and quantification of 73 metabolites in BW25113 and 72 metabolites in Δ*glnP* samples (Table S1), of which 71 were common to both strains. According to the Metabolomics Workbench classification system, the detected metabolites belonged predominantly to amino acids and peptides, tricarboxylic acid (TCA) cycle intermediates, mono- and disaccharides, fatty acids/amines, purines, pyrimidines, and cholines (Table S2).

**Fig. 3:**
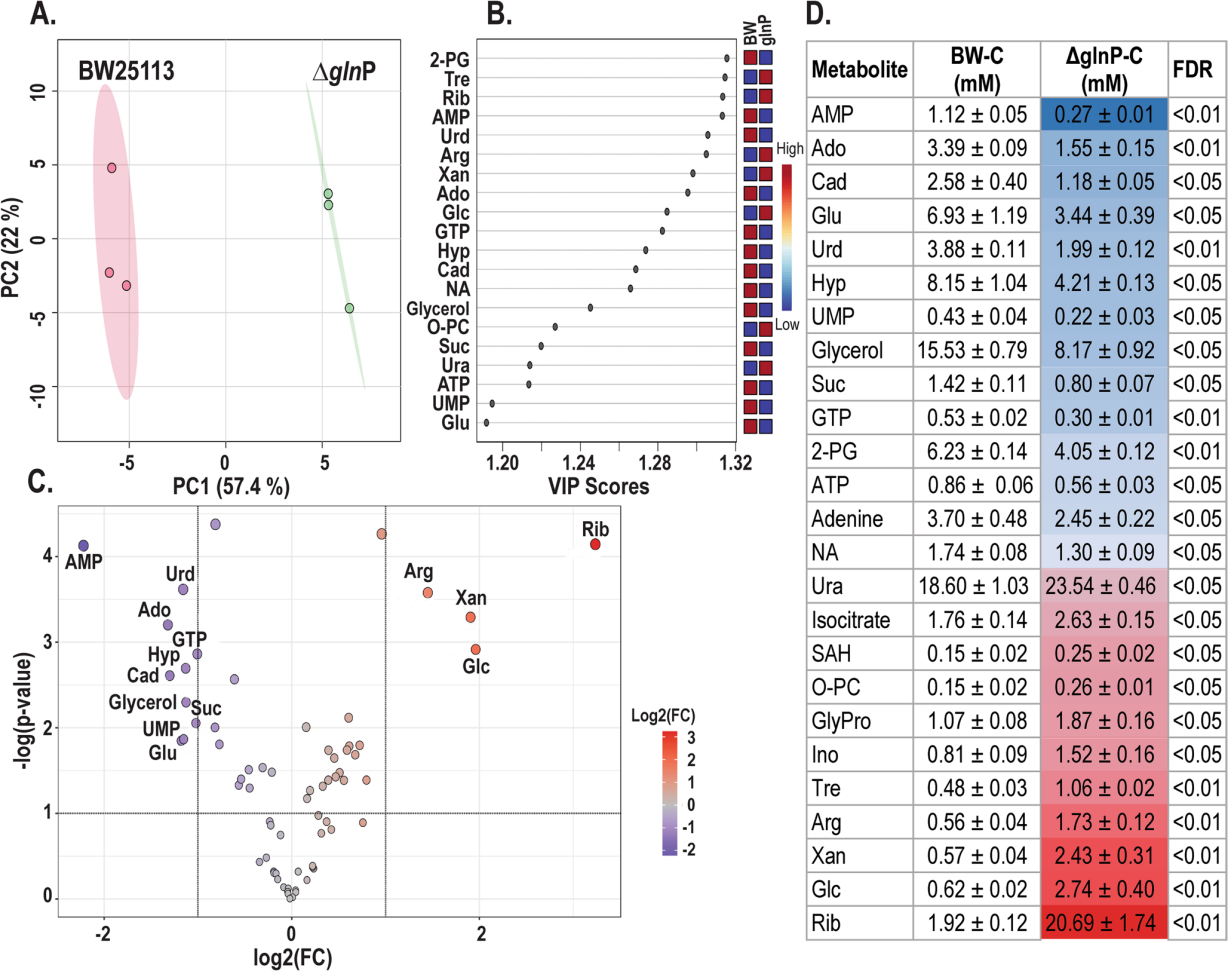
Baseline metabolic differences between *E. coli* BW25113 and Δ*glnP*. (**A**) Principal component analysis (PCA) score plot showing clear separation between wild-type and Δ*glnP* metabolomes under control (untreated) conditions. (**B**) Variable importance in projection (VIP) scores from PLS-DA ranking metabolites contributing most strongly to strain discrimination. (**C**) Volcano plot showing metabolite fold changes (Δ*glnP*/BW25113, log₂ scale) versus – log₁₀ *p*-values. Significantly altered metabolites are highlighted (FDR < 0.05). (**D**) Table summarizing metabolites significantly altered between BW25113 and Δ*glnP* under control conditions, showing mean concentrations (mM) ± standard error and corresponding FDR values. Metabolites are listed in ascending order based on fold-change values in the mutant strain. Cell colors represent a heat map of log_2_-transformed fold changes in metabolite concentrations between BW25113 and Δ*glnP* control groups. Blue indicates decreased and red indicates increased metabolite levels in Δ*glnP* mutant relative to BW25113, and the color intensity reflects the magnitude of the change. Metabolite abbreviations: 2-PG, 2-phosphoglycerate; Ado, adenosine; Arg, arginine; Cad, cadaverine; Glc, glucose; Glu, glutamate; GlyPro, glycylproline; Hyp, hypoxanthine; NA, niacinamide; O-PC, O-phosphocholine; Rib, ribose; SAH, S-adenosylhomocysteine; Suc, sucrose; Tre, trehalose; Ura, uracil; Urd, uridine; Xan, xanthine

To evaluate the effect of *glnP* deletion on the metabolome of BW25113, we first examined baseline metabolic differences between the two strains under untreated conditions. Univariate statistical analysis identified 25 metabolites whose levels were significantly altered in Δ*glnP* mutant compared with the wild-type strain (False Discovery Rate (FDR) adjusted *p* < 0.05) (Fig. 3D). Principal component analysis (PCA) showed separation between BW25113 and the Δ*glnP* mutant along the first principal component (PC1), indicating distinct metabolic profiles between the two strains (Fig. 3A). Metabolites contributing most strongly to strain discrimination in the PLS-DA model included 2-phosphoglycerate, trehalose, ribose, uridine, xanthine, AMP and arginine (Fig. 3B).

Consistent with these results, the volcano plot highlighted significant changes in several metabolites related to nucleotide metabolism (AMP, uridine, xanthine, adenosine), central carbon metabolism (2-phosphoglycerate, glucose), and amino acid metabolism (arginine, glutamate) (Fig. 3C). Together, these results indicate that deletion of *glnP* is associated with broad alterations in the baseline metabolome of *E. coli*. Notably, 12 of the 25 significantly altered metabolites belonged to nucleic acids super class, suggesting that deletion of *glnP* is associated with changes in nucleotide pool composition.

### Impact of BA on the metabolite profiles of *E. coli* BW25113 and Δ*glnP* strains

Having established baseline metabolic differences between the strains, we next examined how BA exposure affects the metabolomes of BW25113 and Δ*glnP* cells. BA treatment resulted in significant alterations in multiple metabolites across both strains, although the magnitude and pattern of these changes differed between BW25113 and the Δ*glnP* mutant. Table 1 summarizes the results of the one-way ANOVA analysis and presents the mean concentrations of quantified metabolites in each BA treatment group, expressed relative to the corresponding control. Only metabolites that displayed significant changes in at least one BA treatment in either strain are included. Metabolites that did not exhibit significant BA-induced changes in either strain– such as 2-hydroxybutyrate, acetate, alanine, caprate, ethanol, glutamate, glycerol, isopropanol, methionine, succinate, threonine, and tyrosine– are omitted from Table 1 but are provided in full in Table S1.

**Table 1:**
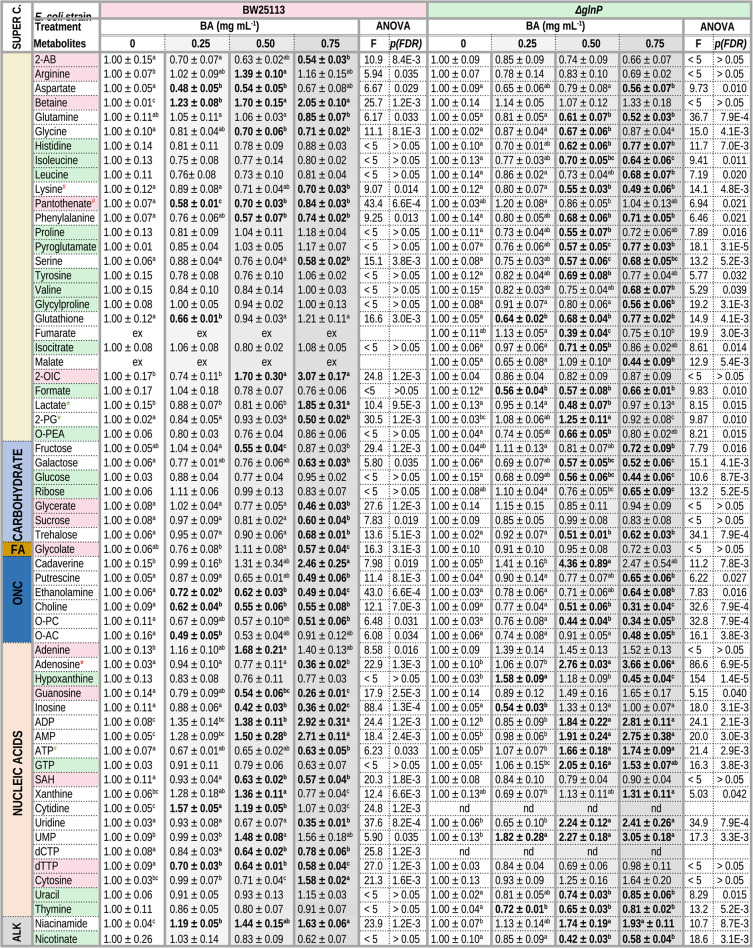
Relative metabolite levels in *E. coli* BW25113 and Δ*glnP* strains exposed to BA

Only metabolites showing a significant change in at least one BA treatment relative to the corresponding control are included. Metabolite concentrations were determined from ^1^H NMR spectra and are expressed as mean ± SE (n = 3) relative to the mean concentration of the untreated control group for each strain (set to 1.00). One-way ANOVA was performed separately for each strain using normalized data. F values represent the ANOVA test statistic reflecting the ratio of between-group to within-group variance, and the corresponding FDR-adjusted *p* values are reported. Different superscript letters (a–c) within a row indicate significant differences among treatment groups according to Tukey’s HSD post-hoc test. Bold values indicate metabolite levels that differ significantly from the corresponding control (*p* < 0.05). “nd” indicates not determined, and “ex” indicates values excluded from the analysis due to high variation among replicates (coefficient of variation > 25%). Metabolite abbreviations: 2-AB, 2-aminobutyrate; 2-OIC, 2-oxoisocaproate; 2-PG, 2-phosphoglycerate; O-AC, O-acetylcholine; O-PC, O-phosphocholine; O-PEA, O-phosphoethanolamine; SAH, S-adenosylhomocysteine. Compounds are classified according to the Metabolomics Workbench chemical taxonomy (super classes): FA, fatty acyls; ONC, organic nitrogen compounds; ALK, alkaloids

* Shapiro-Wilk normality test *p* <0.05 (green: BW25113, red: Δ*glnP*)

# Bartlett test of homogeneity of variances *p* <0.05 (green: BW25113, red: Δ*glnP*)

Out of 73 metabolites detected for BW25113 cells, 42 showed significant changes (FDR < 0.05) at least at one BA concentration (Table 1). In the Δ*glnP* strain, 38 out of 72 detected were significantly altered upon BA treatment. 27 metabolites responded significantly in both strains, although the direction of change differed in some cases. For example, while 0.75 mg mL^−1^ BA caused approximately 3-fold decrease in adenosine and uridine levels in BW25113, the same metabolites increased by about 3-fold in Δ*glnP* strain. 13 metabolites were significantly affected by BA only in wild-type strain (Table 1- pink shading), whereas 18 metabolites responded uniquely in the Δ*glnP* mutant (Table 1- green shading).

In BW25113 cells, BA treatment induced substantial increases in several metabolites associated with amino acid and nucleotide metabolism. At the highest BA concentration (0.75 mg mL^−1^), levels of the branched chain amino acid catabolic intermediate 1-oxoisocaproate, the adenine nucleotides AMP and ADP, and the polyamine cadaverine increased about 2.5-fold or more relative to the control. Moderate increases (approximately 1.5–1.8-fold) were also observed for betaine, cytosine, lactate and niacinamide. Conversely, BA treatment led to pronounced decreases in several nucleosides, amino acids, and metabolites related to central carbon metabolism. Ribonucleosides adenosine, guanosine, inosine and uridine decreased about 3- to 4-fold at highest BA concentration. Several amino acids, including aspartate, glycine, lysine, phenylalanine, and serine also declined by roughly 1.5–2-fold. Additional decreases were observed for metabolites belonging to several biochemical families. These included carbohydrates such as fructose, galactose, sucrose, and trehalose; nucleotides such as ATP, dCTP, and dTTP; and intermediates of central carbon metabolism including glycerate, glycolate, and 2-phosphoglycerate, the latter associated with glycolysis and serine metabolism. BA exposure also reduced levels of several nitrogen containing metabolites, including the polyamine putrescine (derived from arginine and ornithine decarboxylation), choline, O-phosphocholine, and O-acetylcholine (components of phospholipid metabolism), as well as ethanolamine, which can originate from serine decarboxylation. Other decreased metabolites included 2-aminobutyrate, pantothenate, S-adenosylhomocysteine, and glutathione.

In the Δ*glnP* mutant, BA treatment resulted in strong perturbations in nucleotide metabolism. At 0.75 mg mL^−1^ BA, adenosine, ADP, AMP, UMP, and uracil increased > 2.4-fold relative to untreated cells. Additional increases (> 1.25-fold) were observed for 2-phosphoglycerate, ATP, GTP, cadaverine, niacinamide, and xanthine. Hypoxanthine concentration displayed a dose-dependent mixed response, increasing about 1.5-fold at 0.25 mg mL^−1^ BA but decreasing to about half of the control level at 0.75 mg mL^−1^. BA exposure also caused widespread decreases in some metabolite pools. 35 metabolites dropped significantly below control levels in at least one BA treatment group. These included several carbohydrates (glucose, fructose, galactose, ribose, trehalose), organic acids from central carbon metabolism (lactate, malate, isocitrate, fumarate, formate), and numerous amino acids (glutamine, pyroglutamate, glycine, proline, glycylproline, aspartate, histidine, isoleucine, leucine, lysine, tyrosine, phenylalanine, serine, and valine). Several amino acid-derived metabolites were also reduced, including the polyamine putrescine, glutathione, and nitrogen containing compounds such as choline, O-acetylcholine, O-phosphocholine and ethanolamine. Finally, reductions were observed in several nucleosides and nucleobases, including inosine, nicotinate, thymine, and uracil.

### Multivariate analysis of strain and BA treatment effects on the metabolome

**Fig. 4:**
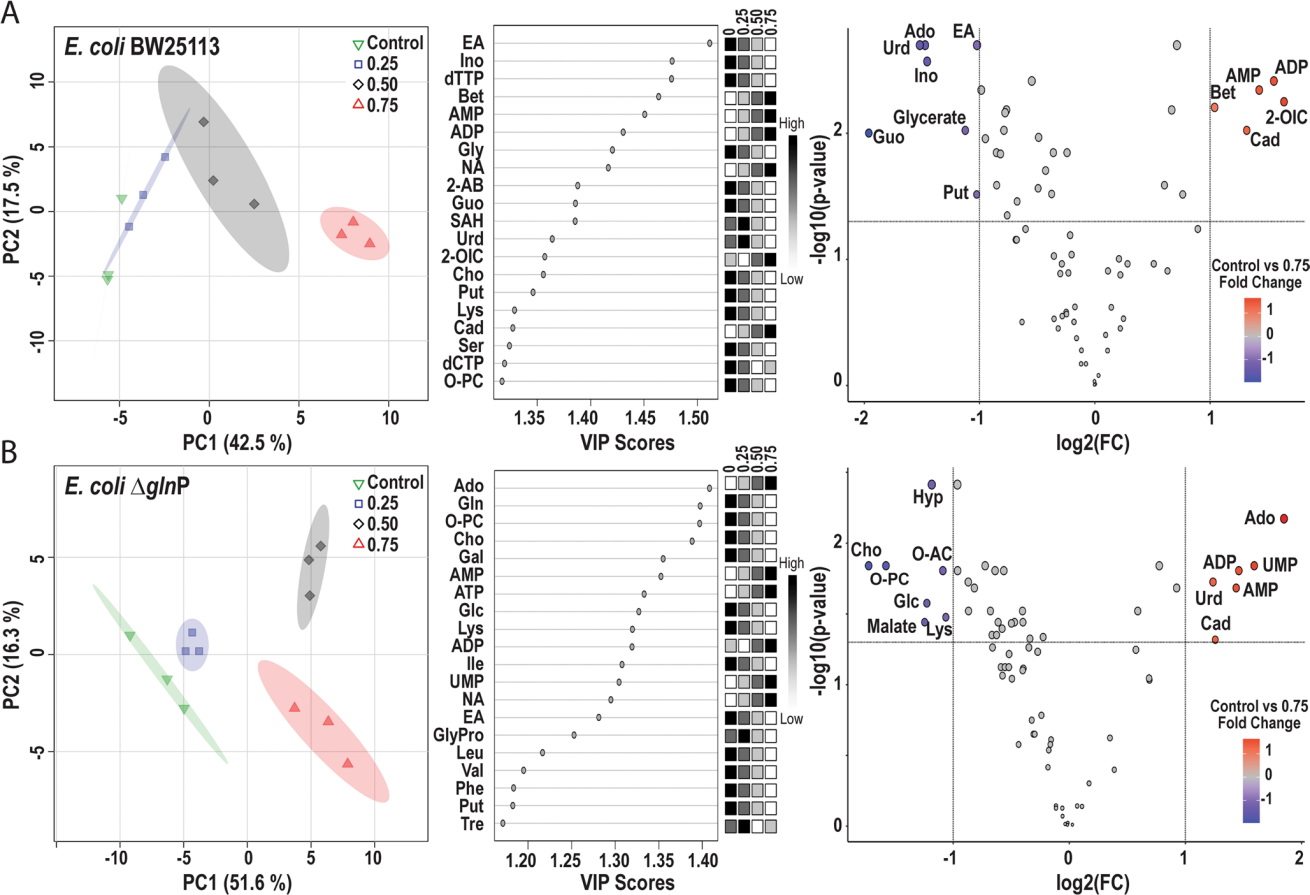
Scores plot obtained from the PCA model of *E. coli* BW25113 (A-left) and Δ*glnP* (B-left) cells treated with BA. The concentration of BA is given on the upright corners of each plot. The shaded areas indicate the 95% confidence regions based on the data points for individual groups. The variable importance in projection (VIP) scores plots of top 20 metabolites obtained from the PLS-DA models (Figure S2) are given in the middle of each panel for BW25113 (A-middle) and Δ*glnP* (B-middle). The shaded boxes on the right side of each VIP scores plot correspond to the relative concentrations of the specific metabolite in each group. Volcano plots of metabolites obtained from analysis of control vs. 0.75 mg mL^− 1^ BA treatment groups with False Discovery Rate adjusted *p* value < 0.05 and fold change > 2.0 cut off value for BW25113 (A-right) and Δ*glnP* (B-right). Metabolite names: 2-AB, 2-aminobutyrate; 2-OIC, 2-oxoisocaproate; Ado, adenosine, Bet, betaine; Cad, cadaverine; Cho, choline; EA, ethanolamine; Gal, galactose; Glc, glucose; Guo; guanosine; Hyp, hypoxanthine; Ino, inosine; NA, niacinamide; O-AC, O-acetylcholine; O-PC, O-phosphocholine; Put, putrescine; SAH, S-adenosylhomocysteine; Tre, trehalose; Urd, uridine

Principal component analysis (PCA) of the BW25113 metabolite dataset showed that BA treatment induced clear metabolic separation at 0.50 and 0.75 mg mL^−1^, whereas the 0.25 mg mL^−1^ treatment partially overlapped with the control group (Fig. 4A, left panel). In contrast, Δ*glnP* cells displayed distinct clustering of the control and each BA treatment group at all concentrations tested (Fig. 4B, left panel). The first three principal components explained 74.5% and 76.6% of the total variance in wild-type and mutant datasets, respectively.

Partial least-squares discriminant analysis (PLS-DA), further confirmed clear separation between control and BA-treated groups for both strains (Figure S2). The models showed high goodness-of-fit (R^2^) and predictive ability (Q^2^) (BW25113: R^2^ = 0.996, Q^2^ = 0.958; Δ*glnP*: R^2^ = 0.995, Q^2^ = 0.926). The first three components explained 68.4% and 74.1% of the total variance in the wild-type and mutant models, respectively. In BW25113, glycine, ethanolamine, betaine, inosine, AMP, ADP, dTTP and niacinamide (VIP > 1.4) contributed most strongly to group separation. In contrast, galactose, adenosine, AMP, choline, O-phosphocholine, and glutamine (VIP > 1.3) were the main discriminating metabolites in Δ*glnP* cells (Fig. 4A and B, middle panels).

Volcano plot analysis comparing control samples with the highest BA treatment highlighted metabolites exhibiting both large fold changes and strong statistical significance (Fig. 4A and B, right panels). AMP, ADP, and cadaverine accumulated in both strains following BA exposure. In BW25113 cells, 2-oxoisocaproate and betaine were also elevated, whereas UMP, uridine, and adenosine increased only in the Δ*glnP* mutant. In contrast, several metabolites declined in response to BA treatment, with strain-specific patterns. In wild-type cells, glycerate, inosine, adenosine, uridine, guanosine, ethanolamine, and putrescine decreased, whereas glucose, malate, lysine, choline, O-acetylcholine, O-phosphocholine, and hypoxanthine declined in Δ*glnP* cells.

**Fig. 5:**
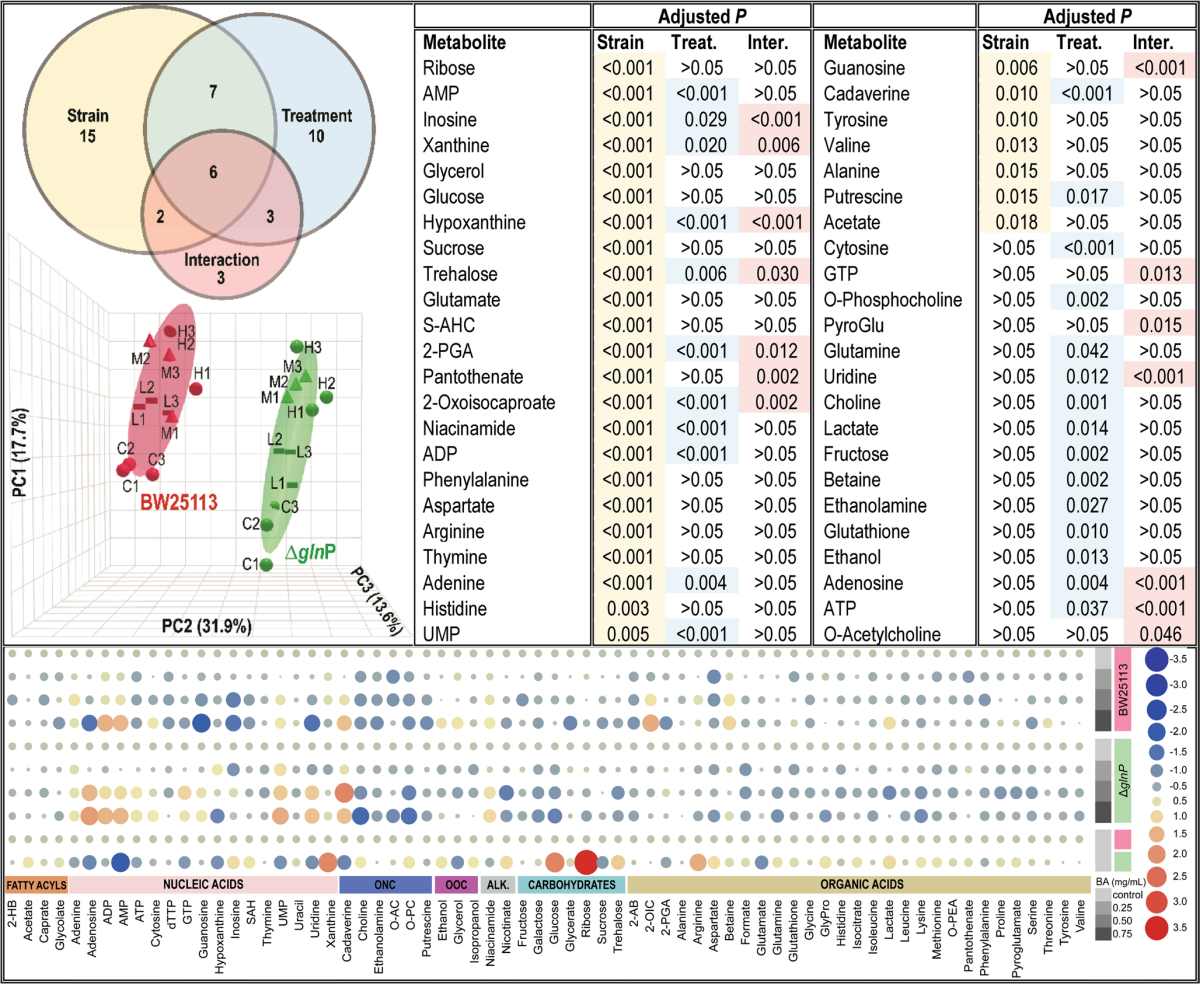
An integrated overview of genotype and BA effects on the *E. coli* metabolome. Venn diagram and PCA scores plot (top-left section) were obtained from a two-way ANOVA analysis of *E. coli* BW25113 (pink) and Δ*glnP* (green) treated with BA. Control group is marked with C in PCA scores plot, and the low, medium, and, high BA treatment groups are annotated as L, M, and H, respectively. Shaded ellipses denote 95% confıdence regions. The percent variance explained by the first three components are shown on each axis. Only those metabolites identified in both strains were included in the analysis. Metabolites displaying a significant difference according to the Bonferroni multiple testing correction (adjusted *p* < 0.05) between the two strains are highlighted in beige, those responsive to treatment are shaded blue, and interaction effects are colored in pink in both two-way ANOVA table (top-right) and Venn diagram. The heatmap (bottom section) displaying the changes in metabolite concentrations upon BA treatment relative to the control for each strain, as well as between control groups of BW25113 and Δ*glnP* (the baseline difference) is drawn using TBtools-II (Chen et al. 2023). The size and the color of the circles indicate the magnitude and the direction of change, respectively

Two-way ANOVA was performed to assess the effects of the Δ*glnP* mutation and BA exposure, and their interaction on metabolite levels. The analysis revealed that metabolite profiles were influenced by both factors, with several metabolites also displaying significant strain × treatment interactions. As shown in Figs. 5 and 30 metabolites exhibited strain-dependent differences after Bonferroni-correction (*p* < 0.05). These differences were particularly evident among sugars, amino acids, and nucleotides, indicating substantial baseline metabolic differences between the two strains independent of BA treatment. For example, ribose, glucose, trehalose, arginine, and xanthine were significantly elevated in untreated Δ*glnP* cells compared with wild-type cells, whereas AMP, adenosine, and cadaverine were reduced (Fig. 5, heatmap; Figure S3, box plots).

In addition, 26 metabolites were significantly associated with BA treatment (*p* < 0.05) (Fig. 5). BA exposure increased the levels of cytosine, adenine, AMP, ADP, UMP, niacinamide, cadaverine, and betaine in both strains, while reducing fructose, trehalose, O-phosphocholine, choline, and hypoxanthine (Figure S3). Several metabolites displayed strain-dependent responses (*p* < 0.01), including inosine, xanthine, hypoxanthine, guanosine, uridine, adenosine, ATP, 2-oxoisocaproate, and pantothenate. Notably, inosine, guanosine, adenosine, uridine, GTP, and ATP levels were oppositely regulated in the two strains, decreasing in wild-type cells but increasing in the Δ*glnP* mutant following BA treatment. These contrasting patterns indicate distinct metabolic adjustments of the two strains to BA exposure and may partly reflect differences in growth dynamics and nucleotide utilization under stress conditions.

ANOVA-simultaneous component analysis (ASCA) was further applied to decompose the effects of mutation, BA treatment, and their interaction on the metabolome. The strain effect was largely driven by ribose (Figure S4), whereas BA-associated variation was mainly explained by ADP, ethanol, O-phosphocholine, choline, ethanolamine, and cytosine. The interaction component was dominated by nucleotides, including adenosine, ATP, guanosine, and uridine, which represented the main discriminating metabolites. Taken together, these complementary analyses indicate that both the Δ*glnP* mutation and BA exposure are associated with changes in central carbon and nucleotide metabolism, several of which show clear strain specificity.

### 6Pathway-level metabolic effects of BA exposure and *glnP* deletion

Pathway analysis was performed to assess the broader metabolic impact of BA exposure and *glnP* deletion in *E. coli*. At 0.25 mg mL^−1^ BA, no pathways were significantly affected in Δ*glnP* after Holm-Bonferroni correction, whereas wild-type cells showed alterations limited to glycerophospholipid metabolism and the one-carbon pool by folate pathway (*p* < 0.05; Table 2 and S3). Increasing BA to 0.50 mg mL^−1^ resulted in convergent alterations in pyrimidine metabolism and the one-carbon pool by folate in both BW25113 (*p* < 0.05) and Δ*glnP* (*p* < 0.01) (Table S4). Additional BW25113-specific changes included purine metabolism and lysine biosynthesis (*p* < 0.05). In Δ*glnP*, significant alterations were observed in folate biosynthesis, riboflavin metabolism, and glutathione metabolism (*p* < 0.01), as well as beta-alanine metabolism and phenylalanine metabolism (*p* < 0.05).

**Table 2:**
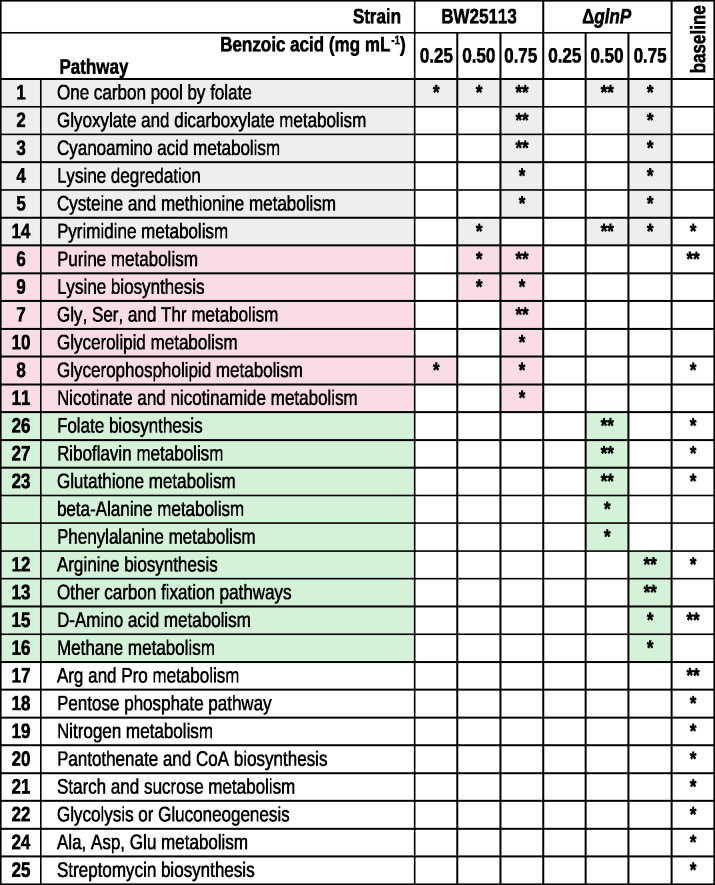
Results of pathway analysis comparing *E. coli* BW25113 and Δ*glnP* strains under control conditions and following treatment with increasing concentrations of BA

Asterisks denote significance based on Holm-Bonferroni adjusted *p* values (* *p* < 0.05, and ** *p* < 0.01). Gray shading highlights pathways commonly affected by BA treatment in both strains, whereas pink and green shading indicate pathways significantly affected only in BW25113 or Δ*glnP*, respectively. Pathways that differ between BW25113 and Δ*glnP* at baseline are also listed (last column)

**Fig. 6:**
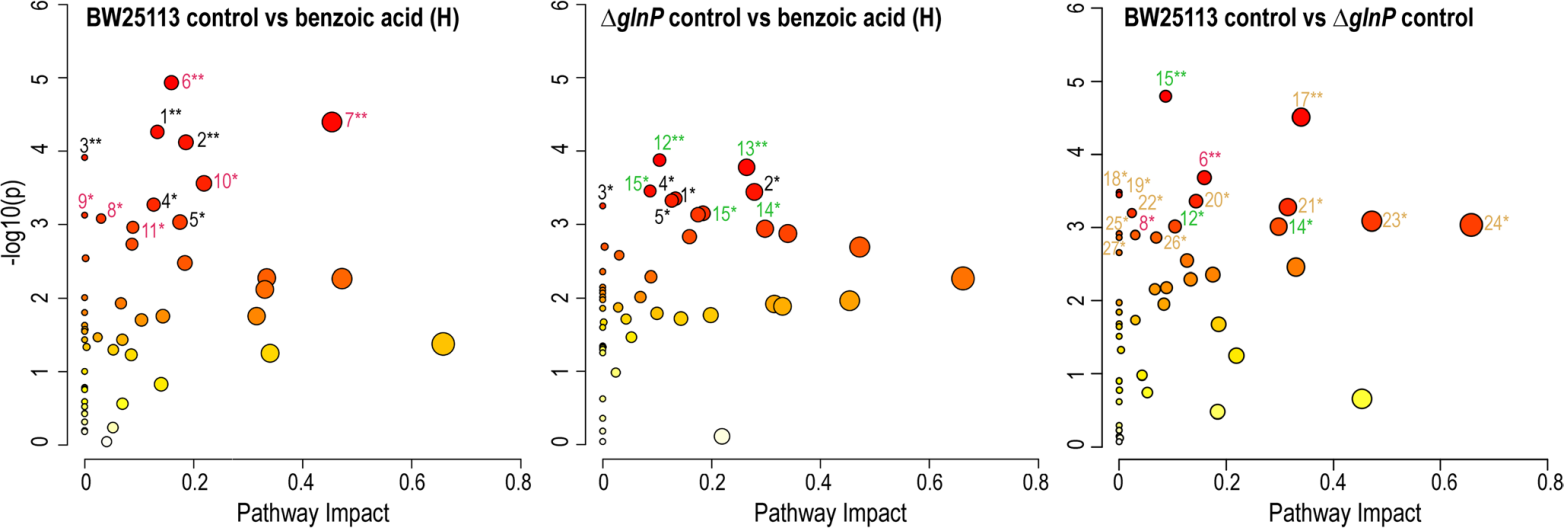
Pathway analysis of *E. coli* BW25113 (wild-type) and Δ*glnP* (mutant) strains. Comparisons include control vs. 0.75 mg mL^−1^ BA (H) treatment groups, as well as wild-type vs. mutant control groups. Circle size indicates the pathway impact, and circle color indicates significance level increasing from yellow to orange to red. the level of significance, respectively. Numbers correspond to pathways listed in Table 2. Asterisks denote significance based on Holm-Bonferroni adjusted *p* values (* *p* < 0.05, and ** *p* < 0.01)

At 0.75 mg mL^−1^ BA, pathway enrichment analysis revealed five pathways altered in both strains–one-carbon pool by folate, glyoxylate and dicarboxylate metabolism, cyanoamino acid metabolism, lysine degradation, and cysteine and methionine metabolism (Fig. 6; Table 2 and S5). Additional strain-specific alterations were also observed. In BW25113 cells, lysine biosynthesis, purine metabolism, glycine, serine and threonine metabolism, glycerophospholipid metabolism, glycerolipid metabolism, and nicotinate and nicotinamide metabolism were significantly affected. In contrast, Δ*glnP* cells showed significant changes in arginine biosynthesis, other carbon fixation pathways, pyrimidine metabolism, D-amino acid metabolism, and methane metabolism.

Notably, several pathways also differed under control conditions (BW25113-C vs. Δ*glnP*-C), with the strongest effects observed in purine metabolism, D-amino acid metabolism, and arginine and proline metabolism (*p* < 0.01) (Fig. 6, right panel). In addition, 13 other pathways were altered at baseline (*p* < 0.05). These results indicate that deletion of *glnP* not only impairs glutamine uptake but is also associated with broader metabolic changes that alter the metabolic state of the mutant strain. Consequently, BA exposure acts on distinct metabolic backgrounds, contributing to the strain-specific responses observed.

## Discussion

This study examined the metabolic responses of *E. coli* to BA exposure using an NMR-based metabolomics approach and investigated the increased sensitivity of the Δ*glnP* mutant lacking the glutamine permease. Although metabolomics cannot directly identify the primary molecular target of BA, it provides a systems-level view of cellular metabolism and reveals pathway-level perturbations associated with stress. The metabolic changes observed in BA-treated cells therefore likely reflect a combination of direct effects of BA and secondary consequences of growth inhibition. Within this framework, our results indicate metabolic signatures consistent with the activation of multiple stress responses, including acid, oxidative, and osmotic stress. Together, these findings provide insight into the physiological state of BA-exposed cells and highlight potential vulnerabilities associated with impaired glutamine transport.

### Effect of *glnP* deletion on BA susceptibility in *E. coli* BW25113

BA exhibits well-established antibacterial activity, although its efficacy varies considerably among bacterial species and even among *E. coli* strains. Reported minimum inhibitory concentrations (MICs) range from 0.375 to > 3.000 mg mL⁻¹ across 57 bacterial species (Seo et al. 2023). For *E. coli*, MIC values typically fall between ~ 1.75 mg mL⁻¹ to 3.125 mg mL⁻¹ depending on the strain (Lin et al. 1996; Burns et al. 2021; Salasar Moghaddam et al. 2024). In our study, the laboratory reference strain BW25113 tolerated BA concentrations up to 1.5 mg mL⁻¹ (Fig. 1), placing its tolerance capacity near the lower end of the reported range for *E. coli*. Beyond its natural form, BA derivatives with enhanced antibacterial potency have also been developed underscoring its versatility as an antimicrobial scaffold (Wei et al. 2018; Li et al. 2021).

Our spot and growth assays show that deletion of *glnP* markedly reduces BA tolerance, lowering the inhibitory threshold from 1.5 mg mL⁻¹ in the wild-type to approximately about 1.0 mg mL⁻¹ in the mutant. Growth curve analysis further revealed that at sublethal BA concentrations (0.50–0.75 mg mL⁻¹), Δ*glnP* cells exhibited prolonged lag phases and substantially slower growth, whereas wild-type cells maintained moderate growth (Fig. 1). These findings indicate that loss of *glnP* sensitizes *E. coli* to BA and suggest that impaired glutamine uptake exacerbates metabolic stress under BA challenge. To investigate the metabolic basis of this phenotype, we performed NMR-based metabolomics, which revealed both shared and strain-specific metabolic response to BA exposure as well as intrinsic baseline differences between BW25113 and Δ*glnP* cells.

### Baseline metabolic differences between BW25113 and Δ*glnP*

NMR-based metabolomic profiling revealed that Δ*glnP* and wild-type *E. coli* cells differ substantially in their baseline metabolite composition (Figs. 3 and 5). Compared with the wild-type, untreated Δ*glnP* cells displayed altered levels of several sugars, amino acids, and nucleotides, indicating that deletion of *glnP* establishes a distinct metabolic state even in the absence of external stress.

One of the most notable differences between the control metabolite profiles of the wild-type and mutant cells was the markedly elevated sugar levels observed in the Δ*glnP* strain (Figure S3). Because *glnP* encodes a key component of the high-affinity glutamine uptake system GlnHPQ, its deletion is expected to perturb cellular nitrogen homeostasis, likely mimicking a nitrogenlimited state. In *E. coli*, nitrogen assimilation and carbon metabolism are tightly coordinated through metabolites such as glutamine, glutamate, and 2-oxoglutarate, which integrate nitrogen availability with central metabolic fluxes (van Heeswijk et al. 2013). Under the conditions where carbon availability exceeds nitrogen assimilation, cells downregulate glycolytic flux and anabolic pathways, diverting excess carbon into soluble sugars, sugar phosphates, and storage polymers, as well as into the synthesis of osmoprotectants such as trehalose, betaine, and proline (Ferneci 1999; van Heeswijk et al. 2013; Chubukov et al. 2017). Beyond serving as a nitrogen source, glutamine also acts as a signaling molecule in the global regulation of nitrogen assimilation and growth rate, adjusting anabolic demand for nitrogen-rich compounds under limiting conditions (Ninfa et al. 2000; Wingreen and Kustu 2001; Forchhammer 2007; Yuan et al. 2009; Chubukov et al. 2014). In this context, the elevated ribose level detected in Δ*glnP* cells may reflect increased flux through the pentose phosphate pathway (PPP) to sustain NADPH generation and to supply ribose precursors for nucleotide salvage, particularly when de novo nucleotide synthesis is constrained (Grucela et al. 2023). Thus, compensatory activation of the PPP may serve as a major outlet for carbon, leading to the accumulation of hexoses and pentoses while maintaining redox balance through NADPH production.

In parallel with these carbon-centered shifts, Δ*glnP* also exhibited broad alterations in nitrogen- and nucleotide-related metabolites. Despite impaired glutamine uptake, intracellular glutamine levels were maintained in the mutant, whereas glutamate was markedly reduced. This may reflect compensatory endogenous glutamine synthesis, potentially through increased reliance on the GS/GOGAT pathway. Such a shift would consume glutamate to sustain intracellular glutamine levels, thereby perturbing the glutamine-glutamate balance that lies at the core of bacterial nitrogen homeostasis (van Heeswijk et al. 2013). In addition, glutamate depletion may also reflect increased utilization by the glutamate decarboxylase (GAD) acid resistance system, in which glutamate is decarboxylated to γ-aminobutyric acid (GABA) while consuming intracellular protons to help maintain cytoplasmic pH under acidic conditions (Li et al. 2024). Because glutamate links nitrogen assimilation to transamination reactions and 2-oxoglutarate-dependent carbon metabolism, this shift could contribute to broader metabolic differences observed in the mutant.

At the same time, the mutant displayed markedly elevated arginine and glycylproline, whereas cadaverine was significantly reduced (Figure S3). Under nitrogen limitation, *E. coli* typically induces the arginine succinyltransferase (AST) pathway to degrade arginine into glutamate and succinate (Schneider et al. 1998). However, the accumulation of arginine together with depleted glutamate in Δ*glnP* may indicate reduced activity of this catabolic route, leading to arginine accumulation rather than utilization – an adaptive response also reported under nutrient limitation and slow-growth conditions (Reitzer 2003).

Elevated glycylproline, a dipeptide commonly associated with peptide turnover during nutrient stress, may likewise reflect increased proteolysis or altered peptide recycling (Minen et al. 2023; Gupta et al. 2024). Meanwhile, decreased cadaverine suggests perturbed polyamine metabolism, which could compromise membrane stability and acid-stress buffering even before BA exposure (DelaVega and Delcour 1995; Samartzidou et al. 2003; Chattopadhyay and Tabor 2013).

Consistent with these alterations in nitrogen metabolism, Δ*glnP* cells also exhibited substantial changes in nucleotide pools. Compared with the wild-type strain, the mutant showed decreased levels of adenine, adenosine, AMP, ATP, GTP, hypoxanthine, UMP and uridine, but elevated levels of inosine, uracil and xanthine, indicating reduced de novo nucleotide synthesis, and compensatory salvage activity (Grove 2025). The concurrent increase in ribose supports enhanced flux through PPP to sustain NADPH and phosphorybosyl-pyrophosphate (PRPP) supply for nucleotide salvage under restricted nitrogen utilization. Collectively, these patterns suggest that loss of *glnP* perturbs the balance between nitrogen assimilation and carbon metabolism, promoting metabolic adjustments that favor nitrogen conservation and nucleotide, amino acid, and polyamine recycling. Such shifts may arise from altered activities of key nitrogen-assimilating enzymes, including GS/GOGAT, and glutamate dehydrogenase (GDH), together with transcriptional changes mediated by nitrogen regulatory protein C (NtrC) (Merrick and Edwards 1995; Zimmer et al. 2000).

### Shared metabolic responses to BA exposure

After establishing distinct baseline metabolic states between the wild-type and Δ*glnP* strains, we examined the metabolic effects of BA exposure in each strain. Two-way ANOVA, multivariate, and pathway analyses revealed both shared and strain-specific metabolic responses (Figs. 5 and 6; Table 2). The common metabolic signature of BA exposure was primarily associated with central carbon metabolism and its coupling to redox balance and folate-mediated one-carbon transfer. Pathways most consistently affected included the one-carbon pool by folate, glyoxylate and dicarboxylate metabolism, and cyanoamino acid metabolism. These observations are consistent with previous studies showing that benzoate and salicylate impose energy and acid stress in *E. coli*, leading to global metabolic adjustments and reconfiguration of central-carbon pathways toward energy conservation (Creamer et al. 2017). Similar slowing of central carbon metabolism and downregulation of cell growth in *E. coli* has been reported as a general strategy during diverse stress responses, including cold, heat, oxidative stress, lactose diauxie, and entry into stationary phase (Jozefczuk et al. 2010). Together, these observations suggest that BA exposure perturbs central carbon utilization, potentially promoting metabolic adjustments that help sustain energy production and redox balance.

Enrichment of lysine degradation among common BA-responsive pathways likely reflects activation of the CadBA lysine decarboxylation system, a well-characterized acid resistance mechanism in *E. coli* (Moreau 2007). Amino-acid decarboxylation reactions contribute to acid tolerance because removal of the carboxyl group consumes intracellular protons and generates alkaline amines, helping counteract cytoplasmic acidification (Foster 2004). Under conditions that promote weak organic-acid accumulation, the *cadBA* operon is induced to decarboxylate lysine to cadaverine while consuming intracellular protons, thereby buffering cytoplasmic pH (Moreau 2007; Li et al. 2024). Because both BW25113 and Δ*glnP* experience intracellular acid stress during BA exposure, engagement of this proton-consuming pathway likely represents a shared adaptive strategy to mitigate acid stress and maintain intracellular pH homeostasis. The decrease in lysine accompanied by increased cadaverine levels observed in both strains are consistent with activation of lysine decarboxylation. Cadaverine may also contribute to stress tolerance by interacting with outer-membrane porins and reducing their permeability to weak acids, thereby stabilizing the membrane (Samartzidou et al. 2003). Similar lysine depletion and cadaverine accumulation have been reported in BW25113 exposed to another phenolic acid, trans-cinnamic acid, supporting a broader role of lysine decarboxylation in weak-acid stress adaptation (Onat-Taşdelen et al. 2024).

Cysteine and methionine metabolism was also significantly affected in both strains during BA challenge, suggesting involvement of redox and one-carbon metabolic processes. In *E. coli*, cysteine serves as the immediate precursor for glutathione (GSH), synthesized by γ-glutamylcysteine synthetase (GshA) and glutathione synthetase (GshB), enzymes central to bacterial redox homeostasis (Fuchs and Warner 1975; Richman and Meister 1975). A *Rhizobium tropici* mutant carrying a Tn5 insertion in a gene highly similar to *E. coli gshB* loses the ability to tolerate weak organic acids as well as oxidative and osmotic stress (Riccillo et al. 2000). The involvement of GSH in acid, osmoadaptive, and oxidative stress responses has also been demonstrated in *E. coli* and *Lactococcus lactis* (Alonso-Moraga et al. 1987; McLaggan et al. 1990; Zhang et al. 2007), underscoring the role of Cys-GSH axis in universal weak-acid, and redox defense among bacteria. Because BA can act both as a weak acid and a pro-oxidant (Piper 1999), increased demand for NADPH-dependent redox buffering is plausible. Enhanced flux through the oxidative PPP could therefore support NADPH production required for GSH regeneration, a response previously observed during oxidative stress in *E. coli* (Christodoulou et al. 2018).

Perturbation of cysteine and methionine metabolism may also reflect increased demand for S-adenosylmethionine (SAM)– the universal methyl donor linking redox control with one-carbon transfer. Methionine-derived SAM has been shown in *Lactiplantibacillus plantarum* to modulate stress-responsive metabolic fluxes, cellular energy distribution, and membrane integrity thereby improving acid resistance (Meng et al. 2022). Consistent with these protective roles, disruption of methionine and SAM biosynthesis sensitizes cells to oxidative stress and leads to rapid cell death in *Mycobacterium tuberculosis*, highlighting the importance of SAM-dependent methylation for bacterial survival (Berney et al. 2015). Similarly, in *Saccharomyces cerevisiae*, methionine supplementation enhances oxidative stress resistance by stimulating flux through the oxidative PPP, thereby increasing NADPH availability for GSH regeneration (Campbell et al. 2016). Thus, the coordinated changes observed in cysteine and methionine metabolism in both strains during BA exposure likely support redox defense, acid tolerance and methylation-dependent stress adaptation pathways.

### Strain-specific metabolic responses to BA exposure

Metabolic analyses revealed pronounced strain-dependent differences in nucleotide metabolism during BA exposure. In wild-type cells, BA treatment was associated with decreased levels of several nucleosides (adenosine, guanosine, inosine, and uridine) and nucleotide triphosphates (ATP and GTP), suggesting reduced nucleotide synthesis and energy charge under stress. In contrast, Δ*glnP* cells exhibited elevated levels of several nucleosides and nucleotide monophosphates, together with altered pools of nucleotide triphosphates (including adenosine, uridine, AMP, ADP, ATP, GTP, and UMP) (Table 1; Fig. 5). This opposing pattern indicates that nucleotides strongly contribute to the strain × treatment interaction and suggests differential remodeling of nucleotide homeostasis under BA stress. Although accumulation of nucleotide intermediates in Δ*glnP* could partly reflect reduced nucleic acid synthesis associated with growth arrest, several nucleotide-related differences were already present under untreated conditions and at lower BA concentrations where growth of both strains was only minimally affected, suggesting that these changes cannot be explained solely by growth inhibition. One interpretation is that wild-type cells reduce de novo nucleotide biosynthesis under stress, potentially through regulatory mechanisms related to the stringent response. In contrast, the Δ*glnP* mutant, which experiences restricted glutamine influx required for de novo purine and pyrimidine biosynthesis, may rely more heavily on nucleotide salvage pathways, leading to accumulation of nucleosides and nucleotide intermediates (Charlier et al. 2018; Zhang et al. 2019; Wang et al. 2020). Consistent with this interpretation, ASCA and two-way ANOVA analyses identified adenosine, guanosine, uridine, and ATP among the strongest contributors to the interaction effect (Figs. 5 and S3), indicating that nucleic acid metabolism represents a major axis of strain-specific metabolic remodeling under BA exposure.

In BW25113, BA exposure uniquely enriched glycerophospholipid and glycerolipid metabolism, consistent with a membrane remodeling response. Weak organic acids are known to increase permeability and reduce stability of *E. coli* membranes (Burns et al. 2021). Mild acid stress has been shown to upregulate *fabA* and *fabB*, leading to increased production of unsaturated fatty acids in exponentially growing *E. coli* to maintain membrane functionality (Xu et al. 2020). In addition, cardiolipin accumulation during stationary phase, osmotic stress, and energy deprivation contributes to stabilization of membrane structures (Hiraoka et al. 1993; Carranza et al. 2017). One reaction that generates glycerol in bacterial lipid metabolism is cardiolipin synthesis, in which two molecules of phosphatidylglycerol condense to form cardiolipin with the release of glycerol (Tan et al. 2012). Interestingly, glycerol levels were consistently lower in Δ*glnP* under both baseline and BA stress conditions (Figs. 5 and S3), which may indicate altered phospholipid remodeling capacity in the mutant strain. However, intracellular glycerol levels alone do not allow direct inference about cardiolipin and phospholipid synthesis, and targeted lipidomic analysis would be required to confirm whether membrane lipid remodeling differs between the strains.

The enrichment of nicotinate and nicotinamide metabolism in BW25113 may reflect increased turnover of NAD^+^/NADP^+^ cofactors required to maintain redox homeostasis. The final step of NAD^+^ biosynthesis, catalyzed by NAD synthetase (NadE), uses glutamine or free ammonia as the nitrogen donor for amidation of nicotinic acid adenine dinucleotide (Schenberger Santos et al. 2020). In *E. coli*, the nitrogen regulatory protein PII directly interacts with NadE to modulate its activity in response to cellular nitrogen and energy status. This regulatory link suggests that sufficient glutamine influx in BW25113 could support NAD^+^ biosynthesis and cofactor turnover during BA challenge. In contrast, restricted glutamine uptake in the Δ*glnP* mutant may limit ammonia availability for NadE activity, potentially constraining NAD^+^ synthesis and redox balancing. Such limitations could weaken induction of nicotinate and nicotinamide metabolism in the mutant and contribute its reduced tolerance to BA.

Although lysine degradation is enriched in both strains during BA exposure, lysine biosynthesis is uniquely enriched in BW25113. This suggests that the wild-type can replenish its lysine pool to sustain the lysine-dependent acid resistance pathway. The observed depletion of aspartate – a key precursor for lysine synthesis – together with stronger cadaverine accumulation in BW25113 (Figure S3) is consistent with active cycling between lysine production and decarboxylation. Lysine decarboxylase (Ldcl/CadA), a key component of this pathway, has been reported to interact with the stringent-response alarmone (p)ppGpp, linking acid resistance mechanism with global metabolic regulation during nutrient stress (Kanjee et al. 2011). Within this framework, the reduced capacity of Δ*glnP* to replenish lysine pools could limit activation of lysine-dependent acid resistance responses to BA, potentially contributing to the increased sensitivity of the mutant strain.

In Δ*glnP*, BA exposure uniquely enriched arginine metabolism, D-amino acid metabolism, other carbon fixation pathways, and methane metabolism, suggesting a distinct strategy to cope with stress under impaired glutamine uptake. Loss of *glnP* likely disrupts the high-glutamate/low-glutamine balance that is essential for nitrogen homeostasis and stress resilience. Perturbations of this balance have been shown to impair growth and render cells hypersensitive under nitrogen-limited conditions (Yan 2007). Consistent with this, Schulz-Mirbach et al. (2022) demonstrated that the *E. coli* amination network is highly flexible only when glutamate–glutamine flux is intact; nitrogen entry at the Glu/Gln node supplies > 90% of cellular nitrogen and governs the capacity of transaminases to redistribute amines throughout metabolism. In our study, Δ*glnP* cells displayed elevated glutamine (~ 1.8-fold) together with reduced glutamate (~ 1.25-fold) compared with BW25113, indicating a disturbed glutamate/glutamine balance. This may reflect compensatory activation of glutamine synthesis via glutamine synthetase (GlnA) when glutamine uptake is impaired (Reitzer 2003; van Heeswijk et al. 2013).Such compensation would preserve glutamine as a nitrogen donor for essential biosynthetic reactions while simultaneously depleting the glutamate pool, consistent with the lower glutamate levels observed in the mutant.

Within this context, enrichment of arginine metabolism in Δ*glnP* during BA exposure may reflect increased reliance on alternative nitrogen- and acid-resistance pathways. In *E. coli*, glutamate-dependent acid resistance (AR2) represents the most effective acid tolerance mechanism and requires a large intracellular glutamate pool (Foster 2004; Aquino et al. 2017; Li et al. 2024). Because Δ*glnP* cells exhibit reduced glutamate levels even under control conditions, their capacity to sustain AR2 cycling may be limited. Under such constraints, the arginine-dependent acid resistance pathway (AR3) may play a greater role in maintaining pH homeostasis. In this system, arginine is decarboxylated to agmatine by arginine decarboxylase while consuming intracellular protons, and agmatine is exchanged for extracellular arginine via the AdiC arginine-agmatine antiporter, thereby contributing to cytoplasmic pH stabilization under acidic conditions (Richard and Foster 2004). This mechanism may help explain the enrichment of arginine metabolism observed in the mutant.

The Δ*glnP* mutant showed unique enrichment of D-amino acid metabolism under BA stress, indicating strain-specific remodeling of cell-wall-associated pathways. D-amino acids such as D-alanine and D-glutamate are essential components of the peptidoglycan and are dynamically regulated to reinforce the cell wall under acidic or membrane-damaging conditions (Lam et al. 2009; Miyamoto and Homma 2021). The selective activation of D-amino acid metabolism in Δ*glnP* under BA stress may therefore reflect compensatory efforts to strengthen the cell envelope when the mutant cannot fully engage energetically demanding phospholipid remodeling pathways. In addition, the reduced baseline glutamate level in the mutant suggests that Δ*glnP* may rely more heavily on racemase-mediated D-amino acid production to maintain peptidoglycan integrity. Together, these observations point to a shift toward cell-wall remodeling as an alternative envelope-stabilizing strategy in the glutamate-limited, glutamine-uptake-deficient mutant. Because the bacterial envelope functions as a permeability barrier, structural changes in the cell wall may influence the diffusion of small molecules across the envelope (Nikaido 2003), including weak organic acids such as BA.

## Conclusions

In summary, our results show that BA induces extensive metabolic remodeling in *E. coli*, affecting central carbon metabolism, redox balance, and acid-resistance pathways. Deletion of *glnP* alters the baseline metabolic state of the cell and reshapes its response to BA stress, particularly in pathways linked to nucleotide metabolism, membrane remodeling, and nitrogen assimilation. These findings suggest that efficient glutamine transport contributes to metabolic resilience under weak-acid stress. Together, the metabolomic data provide a systems-level view of the metabolic adjustments associated with BA exposure and highlight influence of nitrogen metabolism on bacterial stress responses.

## Supplementary information

Below is the link to the electronic supplementary material.

Supplementary Material 1 (DOCX 714 KB)

Supplementary Material 2 (DOCX 212 KB)

Supplementary Material 3 (DOCX 736 KB)

Supplementary Material 4 (DOCX 62.9 KB)

Supplementary Material 5 (DOCX 17.1 KB)

Supplementary Material 6 (DOCX 15.3 KB)

Supplementary Material 7 (DOCX 20.4 KB)

Supplementary Material 8 (DOCX 19.3 KB)

Supplementary Material 9 (DOCX 1.13 MB)

## Data Availability

This study is available at the NIH Common Fund’s National Metabolomics Data Repository (NMDR) website, the Metabolomics Workbench (Sud et al. 2016), [https://www.metabolomicsworkbench.org](http://www.metabolomicsworkbench.org) (NIH grants U2C-DK119886 and OT2-OD030544) where it has been assigned Study ID ST004542. The data can be accessed directly via its Project DOI: [http://dx.doi.org/10.21228/M8G266](http://dx.doi.org/10.21228/M8G266).

## References

[CR1] Aldarini N, Alhasawi AA, Thomas SC, Appanna VD (2017) The role of glutamine synthetase in energy production and glutamine metabolism during oxidative stress. Antonie Van Leeuwenhoek 110:629–639. 10.1007/s10482-017-0829-3 28097538 10.1007/s10482-017-0829-3

[CR2] Alonso-Moraga A, Bocanegra A, Torres JM et al (1987) Glutathione status and sensitivity to GSH-reacting compounds of *Escherichia coli* strains deficient in glutathione metabolism and/or catalase activity. Mol Cell Biochem 73:61–68 3543652 10.1007/BF00229377

[CR3] Aquino P, Honda B, Jaini S et al (2017) Coordinated regulation of acid resistance in *Escherichia coli*. BMC Syst Biol 11:1–15. 10.1186/s12918-016-0376-y 28061857 10.1186/s12918-016-0376-y PMC5217608

[CR4] Baba T, Ara T, Hasegawa M et al (2006) Construction of *Escherichia coli* K-12 in-frame, single-gene knockout mutants: the Keio collection. Mol Syst Biol 2:1–22. 10.1038/msb4100050 10.1038/msb4100050 PMC1681482 16738554

[CR5] Bartlett GR (1948) The inhibition of D-amino acid oxidase by benzoic acid and various monosubstituted benzoic acid derivatives. J Am Chem Soc 70:1010–1011. 10.1021/ja01183a036 18909167 10.1021/ja01183a036

[CR6] Berney M, Berney-Meyer L, Wong KW et al (2015) Essential roles of methionine and S-adenosylmethionine in the autarkic lifestyle of *Mycobacterium tuberculosis*. Proc Natl Acad Sci U S A 112:10008–10013. 10.1073/pnas.1513033112 26221021 10.1073/pnas.1513033112 PMC4538671

[CR7] Bosund I (1963) The action of benzoic and salicylic acids on the metabolism of microorganisms. Adv Food Res 11:331–353. 10.1016/S0065-2628(08)60068-3

[CR8] Brul S, Coote P (1999) Preservative agents in foods: mode of action and microbial resistance mechanisms. Int J Food Microbiol 50:1–17. 10.1016/S0168-1605(99)00072-0 10488839 10.1016/s0168-1605(99)00072-0

[CR9] Burns J, McCoy CP, Irwin NJ (2021) Synergistic activity of weak organic acids against uropathogens. J Hosp Infect 111:78–88. 10.1016/j.jhin.2021.01.024 33545217 10.1016/j.jhin.2021.01.024

[CR10] Campbell K, Vowinckel J, Keller MA, Ralser M (2016) Methionine metabolism alters oxidative stress resistance via the pentose phosphate pathway. Antioxid Redox Signal 24:543–547. 10.1089/ars.2015.6516 26596469 10.1089/ars.2015.6516 PMC4827311

[CR11] Carranza G, Angius F, Ilioaia O et al (2017) Cardiolipin plays an essential role in the formation of intracellular membranes in *Escherichia coli*. Biochimica et Biophysica Acta (BBA) - Biomembranes 1859:1124–1132. 10.1016/j.bbamem.2017.03.006 28284722 10.1016/j.bbamem.2017.03.006

[CR12] Charlier D, Nguyen Le Minh P, Roovers M (2018) Regulation of carbamoylphosphate synthesis in *Escherichia coli*: an amazing metabolite at the crossroad of arginine and pyrimidine biosynthesis. Amino Acids 50:1647–1661. 10.1007/s00726-018-2654-z 30238253 10.1007/s00726-018-2654-z PMC6245113

[CR13] Chattopadhyay MK, Tabor H (2013) Polyamines are critical for the induction of the glutamate decarboxylase-dependent acid resistance system in *Escherichia coli*. J Biol Chem 288:33559–33570. 10.1074/jbc.M113.510552 24097985 10.1074/jbc.M113.510552 PMC3837104

[CR14] Chen C, Wu Y, Li J et al (2023) TBtools-II: A “one for all, all for one” bioinformatics platform for biological big-data mining. Mol Plant 16:1733–1742. 10.1016/j.molp.2023.09.010 37740491 10.1016/j.molp.2023.09.010

[CR15] Chipley JR (2005) Sodium benzoate and benzoic acid. In: Davidson PM, Sofos JN, Branen AL (eds) Antimicrobials in Food, 3rd edn. Taylor & Francis, Boca Raton, FL, pp 11–38

[CR16] Christodoulou D, Link H, Fuhrer T et al (2018) Reserve flux capacity in the pentose phosphate pathway enables *Escherichia coli*’s rapid response to oxidative stress. Cell Syst 6:569–578.e7. 10.1016/j.cels.2018.04.009 29753645 10.1016/j.cels.2018.04.009

[CR17] Chubukov V, Desmarais JJ, Wang G et al (2017) Engineering glucose metabolism of *Escherichia coli* under nitrogen starvation. NPJ Syst Biol Appl 3:1–7. 10.1038/npjsba.2016.35 28725483 10.1038/npjsba.2016.35 PMC5516864

[CR18] Chubukov V, Gerosa L, Kochanowski K, Sauer U (2014) Coordination of microbial metabolism. Nat Rev Microbiol 12:327–340. 10.1038/nrmicro3238 24658329 10.1038/nrmicro3238

[CR19] Creamer KE, Ditmars FS, Basting PJ et al (2017) Benzoate- and salicylate-tolerant strains of *Escherichia coli* K-12 lose antibiotic resistance during laboratory evolution. Appl Environ Microbiol 83:1–19 10.1128/AEM.02736-16 PMC5203621 27793830

[CR20] del Olmo A, Calzada J, Nuñez M (2017) Benzoic acid and its derivatives as naturally occurring compounds in foods and as additives: uses, exposure, and controversy. Crit Rev Food Sci Nutr 57:3084–3103. 10.1080/10408398.2015.1087964 26587821 10.1080/10408398.2015.1087964

[CR21] DelaVega AL, Delcour AH (1995) Cadaverine induces closing of *E. coli* porins. EMBO J 14:6058–6065. 10.1002/j.1460-2075.1995.tb00294.x 8846798 10.1002/j.1460-2075.1995.tb00294.x PMC394726

[CR22] Dibek E, Babayeva A, Elgin ES et al (2025) Genome-wide screen of *Escherichia coli* Keio mutant line identifies genes related to propolis effect. Eur Food Res Technol 251:429–448. 10.1007/s00217-024-04642-5

[CR23] Eklund T (1980) Inhibition of growth and uptake processes in bacteria by some chemical food preservatives. J Appl Bacteriol 48:423–432. 10.1111/j.1365-2672.1980.tb01031.x 6773919 10.1111/j.1365-2672.1980.tb01031.x

[CR24] Elgin ES, Çatav SS, Babayeva A et al (2023) NMR metabolomics analysis of *Escherichia coli* cells treated with Turkish propolis water extract reveals nucleic acid metabolism as the major target. J Appl Microbiol 134:1–14. 10.1093/jambio/lxac031 10.1093/jambio/lxac031 36724215

[CR25] Emwas AH, Roy R, McKay RT et al (2019) NMR spectroscopy for metabolomics research. Metabolites 9:1–39. 10.3390/metabo9070123 10.3390/metabo9070123 PMC6680826 31252628

[CR26] Ferneci T (1999) Regulation by nutrient limitation. Curr Opin Microbiol 2:208–213 10322163 10.1016/S1369-5274(99)80036-8

[CR27] Forchhammer K (2007) Glutamine signalling in bacteria. Front Biosci 12:358–370 17127304 10.2741/2069

[CR28] Foster JW (2004) *Escherichia coli* acid resistance: tales of an amateur acidophile. Nat Rev Microbiol 2:898–907. 10.1038/nrmicro1021 15494746 10.1038/nrmicro1021

[CR29] Freese E, Sheu CW, Galliers E (1973) Function of lipophilic acids as antimicrobial food additives. Nature 241:321–325 4633553 10.1038/241321a0

[CR30] Fuchs JA, Warner HR (1975) Isolation of an *Escherichia coli* mutant deficient in glutathione synthesis. J Bacteriol 124:140–148. 10.1128/jb.129.2.967-972.1977 1100598 10.1128/jb.124.1.140-148.1975 PMC235875

[CR31] Grove A (2025) The delicate balance of bacterial purine homeostasis. Discover Bacteria 2:14. 10.1007/s44351-025-00025-7

[CR32] Grucela PK, Fuhrer T, Sauer U et al (2023) Ribose 5-phosphate: the key metabolite bridging the metabolisms of nucleotides and amino acids during stringent response in *Escherichia coli*? Microbial Cell 10:141–144. 10.15698/mic2023.07.799 37395996 10.15698/mic2023.07.799 PMC10311079

[CR33] Gupta M, Johnson ANT, Cruz ER et al (2024) Global protein turnover quantification in *Escherichia coli* reveals cytoplasmic recycling under nitrogen limitation. Nat Commun 15:5890. 10.1038/s41467-024-49920-8 39003262 10.1038/s41467-024-49920-8 PMC11246515

[CR34] Hazan R, Levine A, Abeliovich H (2004) Benzoic acid, a weak organic acid food preservative, exerts specific effects on intracellular membrane trafficking pathways in *Saccharomyces cerevisiae*. Appl Environ Microbiol 70:4449–4457. 10.1128/AEM.70.8.4449-4457.2004 15294772 10.1128/AEM.70.8.4449-4457.2004 PMC492424

[CR35] Hilton JL (1965) Inhibition of pantothenate biosynthesis by substituted benzoic acids. Weeds 13:267–271

[CR36] Hiraoka S, Matsuzaki H, Shibuya I (1993) Active increase in cardiolipin synthesis in the stationary growth phase and its physiological significance in *Escherichia coli*. FEBS Lett 336:221–224. 10.1016/0014-5793(93)80807-7 8262233 10.1016/0014-5793(93)80807-7

[CR37] Issa HM, Mohammed DH (2025) A critical review on the journey of benzoic acid in the pharmaceutical industry from manufacturing processes through various uses to disposal: an environmental perspective. Environ Anal Health Toxicol 40:1–19. 10.5620/eaht.2025007 10.5620/eaht.2025007 PMC12187383 40400436

[CR38] Jiang L, Sullivan H, Wang B (2022) Principal component analysis (PCA) loading and statistical tests for nuclear magnetic resonance (NMR) metabolomics involving multiple study groups. Anal Lett 55:1648–1662. 10.1080/00032719.2021.2019758

[CR39] Johnson CH, Ivanisevic J, Siuzdak G (2016) Metabolomics: beyond biomarkers and towards mechanisms. Nat Rev Mol Cell Biol 17:451–459. 10.1038/nrm.2016.25 26979502 10.1038/nrm.2016.25 PMC5729912

[CR40] Jozefczuk S, Klie S, Catchpole G et al (2010) Metabolomic and transcriptomic stress response of *Escherichia coli*. Mol Syst Biol 6. 10.1038/msb.2010.18 10.1038/msb.2010.18 PMC2890322 20461071

[CR41] Kanjee U, Gutsche I, Alexopoulos E et al (2011) Linkage between the bacterial acid stress and stringent responses: the structure of the inducible lysine decarboxylase. EMBO J 30:931–944. 10.1038/emboj.2011.5 21278708 10.1038/emboj.2011.5 PMC3049219

[CR42] Kok M, Maton L, Peet MVD et al (2022) Unraveling antimicrobial resistance using metabolomics. Drug Discov Today 27:1774–1783. 10.1016/j.drudis.2022.03.015 35341988 10.1016/j.drudis.2022.03.015

[CR43] Kruk M, Lee JS (1982) Inhibition of *Escherichia coli* timethylamine-N-oxide reductase by food preservatives. J Food Prot 45:241–244. 10.4315/0362-028x-45.3.241 30866283 10.4315/0362-028X-45.3.241

[CR44] Kürkçü MS, Taşdelen KAO, Kabakaş HÖ et al (2025) Genome-wide screen using *Escherichia coli* Keio knockout mutant line reveals genes related to the antimicrobial properties of trans-Cinnamic Acid. World J Microbiol Biotechnol 41:307. 10.1007/s11274-025-04506-4 40781189 10.1007/s11274-025-04506-4

[CR45] Lam H, Oh DC, Cava F et al (2009) D-amino acids govern stationary phase cell wall remodeling in bacteria. Science 325:1552–1555. 10.1126/science.1178123 19762646 10.1126/science.1178123 PMC2759711

[CR46] Lambert LA, Abshire K, Blankenhorn D, Slonczewski JL (1997) Proteins induced in *Escherichia coli* by benzoic acid. J Bacteriol 179:7595–7599 9393730 10.1128/jb.179.23.7595-7599.1997 PMC179716

[CR47] Li Z, Huang Z, Gu P (2024) Response of *Escherichia coli* to acid stress: mechanisms and applications—a narrative review. Microorganisms 12:1774–1786. 10.3390/microorganisms12091774 39338449 10.3390/microorganisms12091774 PMC11434309

[CR48] Li Z, Lin H, Zhou J et al (2021) Synthesis and antimicrobial activity of the hybrid molecules between amoxicillin and derivatives of benzoic acid. Drug Dev Res 82:198–206. 10.1002/ddr.21739 32954547 10.1002/ddr.21739

[CR49] Lin J, Smith MP, Chapin KC et al (1996) Mechanisms of acid resistance in Enterohemorrhagic *Escherichia coli*. Appl Environ Microbiol 62:3094–3100 8795195 10.1128/aem.62.9.3094-3100.1996 PMC168100

[CR50] McLaggan D, Logan TM, Lynn DG, Epstein W (1990) Involvement of γ-glutamyl peptides in osmoadaptation of *Escherichia coli*. J Bacteriol 172:3631–3636. 10.1128/jb.172.7.3631-3636.1990 1972940 10.1128/jb.172.7.3631-3636.1990 PMC213336

[CR51] Meng Q, Li Y, Yuan Y et al (2022) Methionine addition improves the acid tolerance of *Lactiplantibacillus plantarum* by altering cellular metabolic flux, energy distribution, lipids composition. Stress Biol. 10.1007/s44154-022-00072-z 37676340 10.1007/s44154-022-00072-z PMC10441991

[CR52] Merrick MJ, Edwards RA (1995) Nitrogen control in bacteria. Microbiol Rev 59:604–622. 10.1128/mmbr.59.4.604-622.1995 8531888 10.1128/mr.59.4.604-622.1995 PMC239390

[CR53] Minen RI, Thirumalaikumar VP, Skirycz A (2023) Proteinogenic dipeptides, an emerging class of small-molecule regulators. Curr Opin Plant Biol 75:102395. 10.1016/j.pbi.2023.102395 37311365 10.1016/j.pbi.2023.102395

[CR54] Miyakoshi M (2024) Multilayered regulation of amino acid metabolism in *Escherichia coli*. Curr Opin Microbiol 77:102406. 10.1016/j.mib.2023.102406 38061078 10.1016/j.mib.2023.102406

[CR55] Miyamoto T, Homma H (2021) D-amino acid metabolism in bacteria. J BioChem 170:5–13. 10.1093/jb/mvab043 33788945 10.1093/jb/mvab043

[CR56] Moreau PL (2007) The lysine decarboxylase CadA protects *Escherichia coli* starved of phosphate against fermentation acids. J Bacteriol 189:2249–2261. 10.1128/JB.01306-06 17209032 10.1128/JB.01306-06 PMC1899392

[CR57] Nikaido H (2003) Molecular basis of bacterial outer membrane permeability revisited. Microbiol Mol Biol Rev 67:593–656. 10.1128/mmbr.49.1.1-32.1985 14665678 10.1128/MMBR.67.4.593-656.2003 PMC309051

[CR58] Ninfa AJ, Jiang P, Atkinson MR, Peliska JA (2000) Integration of antagonistic signals in the regulation of nitrogen assimilation. In: Stadtman ER, Chock PB (eds) Current Topics in Cellular Regulation. Academic, pp 31–75 10.1016/s0070-2137(01)80002-9 10842746

[CR59] Nishida I, Yanai R, Matsuoid Y et al (2020) Benzoic acid inhibits Coenzyme Q biosynthesis in *Schizosaccharomyces pombe*. PLoS One 15:e0242616. 10.1371/journal.pone.0242616 33232355 10.1371/journal.pone.0242616 PMC7685456

[CR60] Nohno T, Saito T, Hong J (1986) Cloning and complete nucleotide sequence of the *Escherichia coli* glutamine permease operon (glnHPQ). Mol Gen Genet 205:260–269 3027504 10.1007/BF00430437

[CR61] Onat-Taşdelen KA, Öztürkel-Kabakaş H, Yüksektepe E et al (2024) Functional groups matter: metabolomics analysis of *Escherichia coli* exposed to trans-cinnamic acid and its derivatives unveils common and unique targets. World J Microbiol Biotechnol. 10.1007/s11274-023-03841-8 10.1007/s11274-023-03841-8 38114822

[CR62] Pang Z, Lu Y, Zhou G et al (2024) MetaboAnalyst 6.0: towards a unified platform for metabolomics data processing, analysis and interpretation. Nucleic Acids Res 52:398–406. 10.1093/nar/gkae253 10.1093/nar/gkae253 PMC11223798 38587201

[CR63] Pernin A, Guillier L, Dubois-Brissonnet F (2019) Inhibitory activity of phenolic acids against *Listeria monocytogenes*: deciphering the mechanisms of action using three different models. Food Microbiol 80:18–24. 10.1016/j.fm.2018.12.010 30704593 10.1016/j.fm.2018.12.010

[CR64] Piper PW (1999) Yeast superoxide dismutase mutants reveal a pro-oxidant action of weak organic acid food preservatives. Free Radic Biol Med 27:1219–1227. 10.1016/S0891-5849(99)00147-1 10641714 10.1016/s0891-5849(99)00147-1

[CR65] Praphailong W, Fleet GH (1997) The effect of pH, sodium chloride, sucrose, sorbate and benzoate on the growth of food spoilage yeasts. Food Microbiol 14:459–468. 10.1006/fmic.1997.0106

[CR66] Reitzer L (2003) Nitrogen assimilation and global regulation in *Escherichia coli*. Annu Rev Microbiol 57:155–176. 10.1146/annurev.micro.57.030502.090820 12730324 10.1146/annurev.micro.57.030502.090820

[CR67] Riccillo PM, Muglia CI, De Bruijn FJ et al (2000) Glutathione is involved in environmental stress responses in *Rhizobium tropici*, including acid tolerance. J Bacteriol 182:1748–1753. 10.1128/JB.182.6.1748-1753.2000 10692382 10.1128/jb.182.6.1748-1753.2000 PMC94474

[CR68] Richard H, Foster JW (2004) *Escherichia coli* glutamate- and arginine-dependent acid resistance systems increase internal pH and reverse transmembrane potential. J Bacteriol 186:6032–6041. 10.1128/JB.186.18.6032-6041.2004 15342572 10.1128/JB.186.18.6032-6041.2004 PMC515135

[CR69] Richman PG, Meister A (1975) Regulation of γ glutamyl cysteine synthetase by nonallosteric feedback inhibition by glutathione. J Biol Chem 250:1422–1426. 10.1016/s0021-9258(19)41830-9 1112810

[CR70] Salasar Moghaddam F, Tabibian M, Absalan M et al (2024) Comparative analysis of *Escherichia coli* Nissle 1917 ghosts quality: a study of two chemical methods. Arch Microbiol. 10.1007/s00203-024-04095-0 39190149 10.1007/s00203-024-04095-0

[CR71] Salmond CV, Kroll RG, Booth IR (1984) The effect of food preservatives on pH homeostasis in *Escherichia coli*. J Gen Microbiol 130:2845–2850. 10.1099/00221287-130-11-2845 6396375 10.1099/00221287-130-11-2845

[CR72] Samartzidou H, Mehrazin M, Xu Z et al (2003) Cadaverine inhibition of porin plays a role in cell survival at acidic pH. J Bacteriol 185:13–19. 10.1128/JB.185.1.13-19.2003 12486035 10.1128/JB.185.1.13-19.2003 PMC141942

[CR73] Schenberger Santos AR, Marques Gerhardt EC, Parize E et al (2020) NAD+ biosynthesis in bacteria is controlled by global carbon/ nitrogen levels via PII signaling. J Biol Chem 295:6165–6176. 10.1074/jbc.RA120.012793 32179648 10.1074/jbc.RA120.012793 PMC7196632

[CR74] Schneider BL, Kiupakis AK, Reitzer LJ (1998) Arginine catabolism and the arginine succinyltransferase pathway in *Escherichia coli*. J Bacteriol 180:4278–4286. 10.1128/jb.180.16.4278-4286.1998 9696779 10.1128/jb.180.16.4278-4286.1998 PMC107427

[CR75] Schulz-Mirbach H, Müller A, Wu T et al (2022) On the flexibility of the cellular amination network in *E. coli*. eLife 11:1–42. 10.7554/ELIFE.77492 10.7554/eLife.77492 PMC9436414 35876664

[CR76] Seo Y, Sung M, Hwang J, Yoon Y (2023) Minimum inhibitory concentration (MIC) of propionic acid, sorbic acid, and benzoic acid against food spoilage microorganisms in animal products to use MIC as threshold for natural preservative production. Food Sci Anim Resour 43:319–330. 10.5851/kosfa.2022.e79 36909850 10.5851/kosfa.2022.e79 PMC9998193

[CR77] Sheu CW, Salomon D, Simmons JL et al (1975) Inhibitory effects of lipophilic acids and related compounds on bacteria and mammalian cells. Antimicrob Agents Chemother 7:349–363. 10.1128/AAC.7.3.349 1137388 10.1128/aac.7.3.349 PMC429138

[CR78] Sieber R, Bütikofer U, Bosset JO (1995) Benzoic acid as a natural compound in cultured dairy products and cheese. Int Dairy J 5:227–246. 10.1016/0958-6946(94)00005-A

[CR79] Smilde AK, Jansen JJ, Hoefsloot HCJ et al (2005) ANOVA-simultaneous component analysis (ASCA): a new tool for analyzing designed metabolomics data. Bioinformatics 21:3043–3048. 10.1093/bioinformatics/bti476 15890747 10.1093/bioinformatics/bti476

[CR80] Sud M, Fahy E, Cotter D et al (2016) Metabolomics workbench : an international repository for metabolomics data and metadata, metabolite standards, protocols, tutorials and training, and analysis tools. Nucleic Acids Res 44:463–470. 10.1093/nar/gkv1042 10.1093/nar/gkv1042 PMC4702780 26467476

[CR81] Tan BK, Bogdanov M, Zhao J et al (2012) Discovery of a cardiolipin synthase utilizing phosphatidylethanolamine and phosphatidylglycerol as substrates. Proc Natl Acad Sci U S A 109:16504–16509. 10.1073/pnas.1212797109 22988102 10.1073/pnas.1212797109 PMC3478633

[CR82] van Heeswijk WC, Westerhoff HV, Boogerd FC (2013) Nitrogen assimilation in *Escherichia coli*: putting molecular data into a systems perspective. Microbiol Mol Biol Rev 77:628–695. 10.1128/mmbr.00025-13 24296575 10.1128/MMBR.00025-13 PMC3973380

[CR83] Wang B, Grant RA, Laub MT (2020) ppGpp coordinates nucleotide and amino-acid synthesis in *E. coli* during starvation. Mol Cell 80:29–42.e10. 10.1016/j.molcel.2020.08.005 32857952 10.1016/j.molcel.2020.08.005 PMC8362273

[CR84] Warth AD (1991) Effect of benzoic acid on glycolytic metabolite levels and intracellular pH in *Saccharomyces cerevisiae*. Appl Environ Microbiol 57:3415–3417. 10.1128/aem.57.12.3415-3417.1991a 1785917 10.1128/aem.57.12.3415-3417.1991 PMC183989

[CR85] Warth AD (1991b) Mechanism of action of benzoic acid on Zygosaccharomyces bailii: Effects on glycolytic metabolite levels, energy production and intracellular pH. Appl Environ Microbiol 57:1–4 1785916 10.1128/aem.57.12.3410-3414.1991 PMC183988

[CR86] Wei Q, Wang X, Cheng JH et al (2018) Synthesis and antimicrobial activities of novel sorbic and benzoic acid amide derivatives. Food Chem 268:220–232. 10.1016/j.foodchem.2018.06.071 30064751 10.1016/j.foodchem.2018.06.071

[CR87] Wingreen NS, Kustu S (2001) Coordinated slowing of metabolism in enteric bacteria under nitrogen limitation. A perspective

[CR88] Xia J, Wishart DS (2011) Web-based inference of biological patterns, functions and pathways from metabolomic data using MetaboAnalyst. Nat Protoc 6:743–760. 10.1038/nprot.2011.319 21637195 10.1038/nprot.2011.319

[CR89] Xu Y, Zhao Z, Tong W et al (2020) An acid-tolerance response system protecting exponentially growing *Escherichia coli*. Nat Commun 11:1496. 10.1038/s41467-020-15350-5 32198415 10.1038/s41467-020-15350-5 PMC7083825

[CR90] Yan D (2007) Protection of the glutamate pool concentration in enteric bacteria. Proc Natl Acad Sci USA 104:9475–9480. 10.1073/pnas.0703360104 17517610 10.1073/pnas.0703360104 PMC1890519

[CR91] Yuan J, Doucette CD, Fowler WU et al (2009) Metabolomics-driven quantitative analysis of ammonia assimilation in *E. coli*. Mol Syst Biol. 10.1038/msb.2009.60 19690571 10.1038/msb.2009.60 PMC2736657

[CR92] Zhang J, Fu RY, Hugenholtz J et al (2007) Glutathione protects *Lactococcus lactis* against acid stress. Appl Environ Microbiol 73:5268–5275. 10.1128/AEM.02787-06 17601814 10.1128/AEM.02787-06 PMC1950978

[CR93] Zhang YE, Bærentsen RL, Fuhrer T et al (2019) (p)ppGpp regulates a bacterial nucleosidase by an allosteric two-domain switch. Mol Cell 74:1239-1249e.e4. 10.1016/j.molcel.2019.03.035 31023582 10.1016/j.molcel.2019.03.035

[CR94] Zimmer DP, Soupene E, Lee HL et al (2000) Nitrogen regulatory protein C-controlled genes of *Escherichia coli*: scavenging as a defense against nitrogen limitation. Proc Natl Acad Sci U S A 97:14674–14679. 10.1073/pnas.97.26.14674 11121068 10.1073/pnas.97.26.14674 PMC18977

