# Supplementary material for: Metabolic responses to benzoic acid stress and glutamine transport-dependent vulnerabilities in Escherichia coli revealed by NMR metabolomics
Source: World J Microbiol Biotechnol. 2026 Apr 24;42(5):230. doi: 10.1007/s11274-026-04971-5 (PMC13106250; doi:10.1007/s11274-026-04971-5)
Supplement: Supplementary file 1 — Supplementary Material 1 (DOCX 714 KB) [file 11274_2026_4971_MOESM1_ESM.docx]

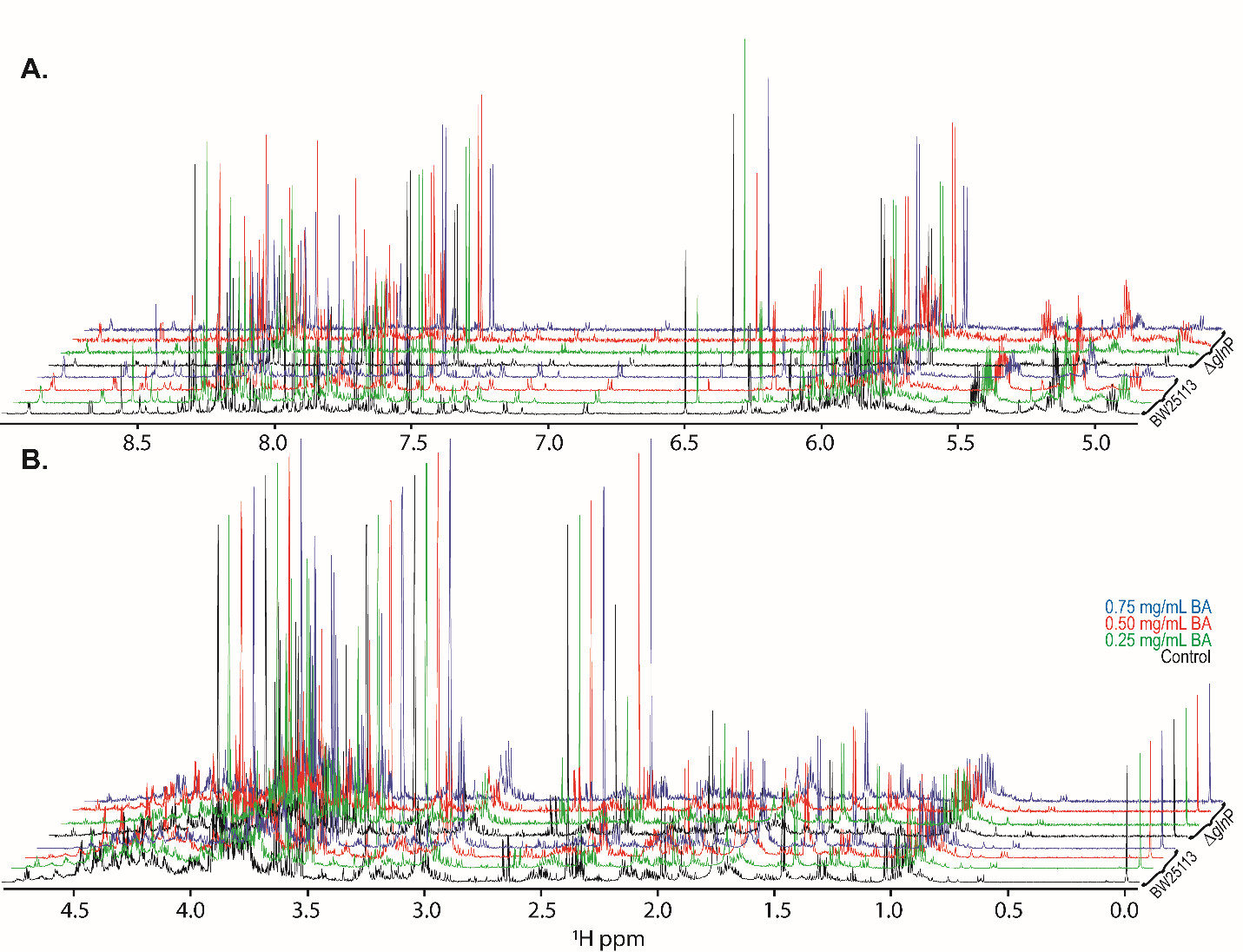


**Figure S1.** Representative ^1^H-NMR spectra of polar metabolite extracts from *E. coli* BW25113 (wild-type) and *ΔglnP* cultured in LB medium in the absence and presence of the various concentrations of benzoic acid (BA). **A**. Downfield region (9.0-5.0 ppm). **B.** Upfield region (4.5-0.0 ppm). For each strain, spectra from control (black), and BA-treated cultures—0.25 mg/mL (green), 0.50 mg/mL (red), and 0.75 mg/mL (blue)—are overlaid and vertically offset for clarity.
