# Supplementary material for: Metabolic responses to benzoic acid stress and glutamine transport-dependent vulnerabilities in Escherichia coli revealed by NMR metabolomics
Source: World J Microbiol Biotechnol. 2026 Apr 24;42(5):230. doi: 10.1007/s11274-026-04971-5 (PMC13106250; doi:10.1007/s11274-026-04971-5)
Supplement: Supplementary file 2 — Supplementary Material 2 (DOCX 212 KB) [file 11274_2026_4971_MOESM2_ESM.docx]

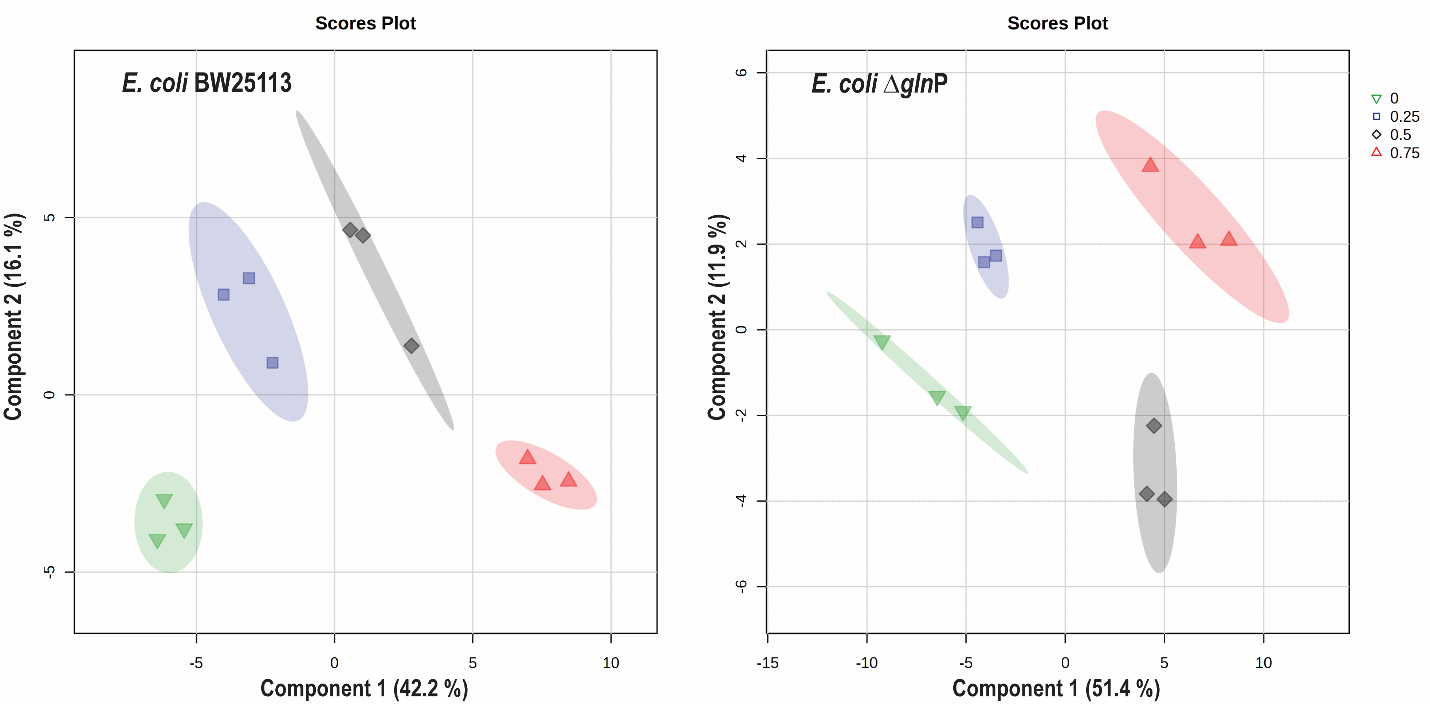


**Figure S2.** PLS-DA scores plots of ^1^H-NMR metabolite profiles from E. coli BW25113 (left) and ΔglnP (right) cultured in LB and exposed to benzoic acid (BA). Colors/shapes denote BA dose: 0 mg/mL (green inverted triangles), 0.25 mg/mL (blue squares), 0.50 mg/mL (gray dimonds), and 0.75 mg/mL (red triangles). Points are biological replicates; shaded ellipses indicate 95% confidence regions. Model quality statistics (R² and cross-validated Q²) are reported in the Results.
