# Supplementary material for: Metabolic responses to benzoic acid stress and glutamine transport-dependent vulnerabilities in Escherichia coli revealed by NMR metabolomics
Source: World J Microbiol Biotechnol. 2026 Apr 24;42(5):230. doi: 10.1007/s11274-026-04971-5 (PMC13106250; doi:10.1007/s11274-026-04971-5)
Supplement: Supplementary file 3 — Supplementary Material 3 (DOCX 736 KB) [file 11274_2026_4971_MOESM3_ESM.docx]

1. ­­ B. C.


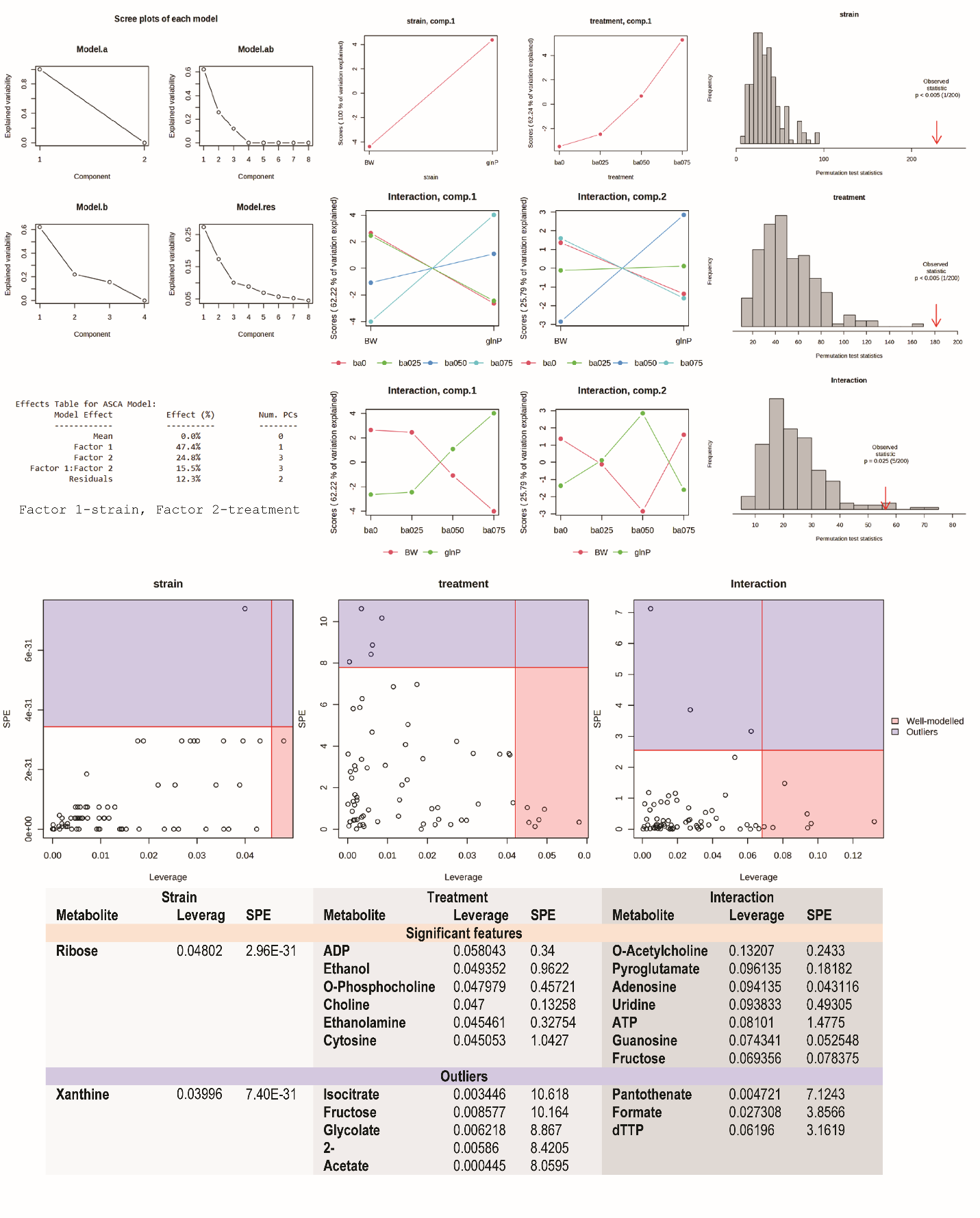


D.

**Figure S4: ASCA analysis of metabolite profiles in** E. coli **BW25113 and ΔglnP cells under benzoic acid stress. A.** Model validation plots showing variance explained for strain, treatment, and interaction effects compared to permutation tests. Strain effect was primarily captured in component 1, treatment effect in component 1, and interaction effects across components 1 and 2. **B.** Score plots illustrating separation of wild-type (BW25113) and Δ*glnP* cells (strain effect), dose-dependent clustering of benzoic acid treatments (treatment effect), and distinct genotype-specific responses across benzoic acid concentrations (interaction effects). **C.** Model validations through permutations for the strain, treatment and interaction **D.** Leverage vs. SPE (squared prediction error) plots for strain, treatment, and interaction models, highlighting well-modeled features (pink) and outliers (purple).
