# Supplementary material for: Metabolic responses to benzoic acid stress and glutamine transport-dependent vulnerabilities in Escherichia coli revealed by NMR metabolomics
Source: World J Microbiol Biotechnol. 2026 Apr 24;42(5):230. doi: 10.1007/s11274-026-04971-5 (PMC13106250; doi:10.1007/s11274-026-04971-5)
Supplement: Supplementary file 4 — Supplementary Material 4 (DOCX 62.9 KB) [file 11274_2026_4971_MOESM4_ESM.docx]

**Table S1.** The list of metabolites determined in ^1^H NMR spectra of untreated (control) and benzoic acid (BA) treated wild type (BW25113) and mutant (Δ*glnP*) *E. coli* cells. Concentrations are given as mean ± SE (n=3) relative to the mean concentration of the control group. FDR (False Discovery Rate) adjusted *p*-values of <0.05 are listed for one-way ANOVA carried out using normalized data. Different superscript letters (^a^, ^b^, and ^c^) in the same line within strain show the significant differences in the metabolite concentrations according to Tukey’s Honestly Significant Difference post-hoc analysis. Bold numbers indicate that the concentration of the metabolite in the treatment group differs significantly from the corresponding control concentration (*p* <0.05). “nd” and “ex” mean not determined and excluded from the analysis due to high variation within replicates (coefficient of variation >25%), respectively. FA: Fatty Acyls, ONC: Organic Nitrogen compounds, OOC: Organic Oxygen Compounds, AK: Alkaloids.

|  | ***E. coli* strain** | **BW25113** | | | | | | ***Δgln*P** | | | | | |
| --- | --- | --- | --- | --- | --- | --- | --- | --- | --- | --- | --- | --- | --- |
|  | **Treatment** | **Benzoic Acid (mg.mL^-1^)** | | | | **ANOVA** | | **Benzoic Acid (mg.mL^-1^)** | | | | **ANOVA** | |
|  | **Metabolites** | **0** | **0.25** | **0.50** | **0.75** | **F** | ***p(FDR)*** | **0** | **0.25** | **0.50** | **0.75** | **F** | ***p(FDR)*** |
| **ORGANIC ACIDS** | **2-Aminobutyrate** | 1.00 ± 0.15^a^ | 0.70 ± 0.07^a^ | 0.63 ± 0.02^ab^ | **0.54 ± 0.03^b^** | 10.9 | 8.44E-3 | 1.00 ± 0.09 | 0.85 ± 0.09 | 0.74 ± 0.09 | 0.66 ± 0.07 | < 5 | > 0.05 |
|  | **Alanine** | 1.00 ± 0.08 | 0.87 ± 0.07 | 0.80 ± 0.08 | 1.09 ± 0.05 | < 5 | > 0.05 | 1.00 ± 0.07 | 0.94 ± 0.12 | 0.90 ± 0.08 | 1.01 ± 0.07 | < 5 | > 0.05 |
|  | **Arginine** | 1.00 ± 0.07^b^ | 1.02 ± 0.09^ab^ | **1.39 ± 0.10^a^** | 1.16 ± 0.15^ab^ | 5.94 | 0.0346 | 1.00 ± 0.07 | 0.78 ± 0.14 | 0.83 ± 0.10 | 0.69 ± 0.02 | < 5 | > 0.05 |
|  | **Aspartate** | 1.00 ± 0.05^a^ | **0.48 ± 0.05^b^** | **0.54 ± 0.05^b^** | 0.67 ± 0.08^ab^ | 6.67 | 0.0291 | 1.00 ± 0.09^a^ | 0.65 ± 0.06^ab^ | 0.79 ± 0.08^a^ | **0.56 ± 0.07^b^** | 9.73 | 0.0103 |
|  | **Betaine** | 1.00 ± 0.01^c^ | **1.23 ± 0.08^b^** | **1.70 ± 0.15^a^** | **2.05 ± 0.10^a^** | 25.7 | 1.21E-3 | 1.00 ± 0.14 | 1.14 ± 0.05 | 1.07 ± 0.12 | 1.33 ± 0.18 | < 5 | > 0.05 |
|  | **Glutamate** | 1.00 ± 0.17 | 1.07 ± 0.14 | 0.83 ± 0.14 | 0.78 ± 0.09 | < 5 | > 0.05 | 1.00 ± 0.11 | 1.04 ± 0.04 | 0.85 ± 0.04 | 1.10 ± 0.14 | < 5 | > 0.05 |
|  | **Glutamine** | 1.00 ± 0.11^ab^ | 1.05 ± 0.11^a^ | 1.06 ± 0.03^a^ | **0.85 ± 0.07^b^** | 6.17 | 0.0332 | 1.00 ± 0.05^a^ | 0.81 ± 0.05^a^ | **0.61 ± 0.07^b^** | **0.52 ± 0.03^b^** | 36.7 | 7.88E-4 |
|  | **Glycine** | 1.00 ± 0.10^a^ | 0.81 ± 0.04^ab^ | **0.70 ± 0.06^b^** | **0.71 ± 0.02^b^** | 11.1 | 8.08E-3 | 1.00 ± 0.02^a^ | 0.87 ± 0.04^a^ | **0.67 ± 0.06^b^** | 0.87 ± 0.04^a^ | 15.0 | 4.13E-3 |
|  | **Histidine** | 1.00 ± 0.14 | 0.81 ± 0.11 | 0.78 ± 0.09 | 0.88 ± 0.03 | < 5 | > 0.05 | 1.00 ± 0.10^a^ | 0.70 ± 0.01^ab^ | **0.62 ± 0.06^b^** | **0.77 ± 0.07^b^** | 11.7 | 7.02E-3 |
|  | **Isoleucine** | 1.00 ± 0.13 | 0.75 ± 0.08 | 0.77 ± 0.14 | 0.80 ± 0.02 | < 5 | > 0.05 | 1.00 ± 0.13^a^ | 0.77 ± 0.03^ab^ | **0.70 ± 0.05^bc^** | **0.64 ± 0.06^c^** | 9.41 | 0.0111 |
|  | **Leucine** | 1.00 ± 0.11 | 0.76± 0.08 | 0.73 ± 0.10 | 0.81 ± 0.04 | < 5 | > 0.05 | 1.00 ± 0.14^a^ | 0.86 ± 0.02^a^ | 0.73 ± 0.04^ab^ | **0.68 ± 0.07^b^** | 7.19 | 0.0197 |
|  | **Lysine^#^** | 1.00 ± 0.12^a^ | 0.89 ± 0.08^a^ | 0.71 ± 0.04^ab^ | **0.70 ± 0.03^b^** | 9.07 | 0.0136 | 1.00 ± 0.12^a^ | 0.80 ± 0.07^a^ | **0.55 ± 0.03^b^** | **0.49 ± 0.06^b^** | 14.1 | 4.79E-3 |
|  | **Methionine** | 1.00 ± 0.07 | 0.68 ± 0.05 | 0.85 ± 0.08 | 0.88 ± 0.07 | < 5 | > 0.05 | 1.00 ± 0.12 | 0.69 ± 0.01 | 0.76 ± 0.06 | 0.69 ± 0.08 | < 5 | > 0.05 |
|  | **Pantothenate^#^** | 1.00 ± 0.07^a^ | **0.58 ± 0.01^c^** | **0.70 ± 0.03^b^** | **0.84 ± 0.03^b^** | 43.4 | 6.61E-4 | 1.00 ± 0.03^ab^ | 1.20 ± 0.08^a^ | 0.86 ± 0.05^b^ | 1.04 ± 0.13^ab^ | 6.94 | 0.0213 |
|  | **Phenylalanine** | 1.00 ± 0.07^a^ | 0.76 ± 0.06^ab^ | **0.57 ± 0.07^b^** | **0.74 ± 0.02^b^** | 9.25 | 0.0132 | 1.00 ± 0.14^a^ | 0.80 ± 0.05^ab^ | **0.68 ± 0.06^b^** | **0.71 ± 0.05^b^** | 6.46 | 0.0212 |
|  | **Proline** | 1.00 ± 0.13 | 0.81 ± 0.09 | 1.04 ± 0.11 | 1.18 ± 0.04 | < 5 | > 0.05 | 1.00 ± 0.11^a^ | 0.73 ± 0.04^ab^ | **0.55 ± 0.07^b^** | 0.72 ± 0.06^ab^ | 7.89 | 0.0161 |
|  | **Pyroglutamate** | 1.00 ± 0.01 | 0.85 ± 0.04 | 1.03 ± 0.05 | 1.17 ± 0.07 | < 5 | > 0.05 | 1.00 ± 0.07^a^ | 0.76 ± 0.06^ab^ | **0.57 ± 0.05^c^** | **0.77 ± 0.03^b^** | 18.1 | 3.05E-5 |
|  | **Serine** | 1.00 ± 0.06^a^ | 0.88 ± 0.04^a^ | 0.76 ± 0.04^a^ | **0.58 ± 0.02^b^** | 15.1 | 3.75E-3 | 1.00 ± 0.08^a^ | 0.75 ± 0.03^ab^ | **0.57 ± 0.06^c^** | **0.68 ± 0.05^bc^** | 13.2 | 5.19E-3 |
|  | **Threonine** | 1.00 ± 0.08 | 0.83 ± 0.05 | 0.86 ± 0.12 | 1.44 ± 0.14 | < 5 | > 0.05 | 1.00 ± 0.07 | 0.84 ± 0.08 | 0.87 ± 0.10 | 0.81 ± 0.10 | < 5 | > 0.05 |
|  | **Tyrosine** | 1.00 ± 0.15 | 0.78 ± 0.08 | 0.76 ± 0.10 | 1.06 ± 0.02 | < 5 | > 0.05 | 1.00 ± 0.12^a^ | 0.82 ± 0.04^ab^ | **0.69 ± 0.08^b^** | 0.77 ± 0.04^ab^ | 5.77 | 0.0320 |
|  | **Valine** | 1.00 ± 0.15 | 0.84 ± 0.10 | 0.84 ± 0.14 | 1.00 ± 0.03 | < 5 | > 0.05 | 1.00 ± 0.15^a^ | 0.82 ± 0.03^ab^ | 0.75 ± 0.04^ab^ | **0.68 ± 0.07^b^** | 5.29 | 0.0388 |
|  | **Glycylproline** | 1.00 ± 0.08 | 1.00 ± 0.05 | 0.94 ± 0.02 | 1.00 ± 0.13 | < 5 | > 0.05 | 1.00 ± 0.08^a^ | 0.91 ± 0.07^a^ | 0.80 ± 0.06^a^ | **0.56 ± 0.06^b^** | 19.2 | 3.05E-3 |
|  | **Glutathione** | 1.00 ± 0.12^a^ | **0.66 ± 0.01^b^** | 0.94 ± 0.03^a^ | 1.21 ± 0.11^a^ | 16.6 | 3.01E-3 | 1.00 ± 0.05^a^ | **0.64 ± 0.02^b^** | **0.68 ± 0.04^b^** | **0.77 ± 0.02^b^** | 14.9 | 4.13E-3 |
|  | **Fumarate** | ex | ex | ex | ex |  |  | 1.00 ± 0.11^ab^ | 1.13 ± 0.05^a^ | **0.39 ± 0.04^c^** | 0.75 ± 0.10^b^ | 19.9 | 2.95E-3 |
|  | **Isocitrate** | 1.00 ± 0.08 | 1.06 ± 0.08 | 0.80 ± 0.02 | 1.08 ± 0.05 | < 5 | > 0.05 | 1.00 ± 0.06^a^ | 0.97 ± 0.06^a^ | **0.71 ± 0.05^b^** | 0.86 ± 0.02^ab^ | 8.61 | 0.0140 |
|  | **Malate** | ex | ex | ex | ex |  |  | 1.00 ± 0.05^a^ | 0.65 ± 0.08^a^ | 1.09 ± 0.10^a^ | **0.44 ± 0.09^b^** | 12.9 | 5.37E-3 |
|  | **Succinate** | 1.00 ± 0.21 | 0.84 ± 0.10 | 0.71 ± 0.11 | 0.97 ± 0.05 | < 5 | > 0.05 | ex | ex | ex | ex |  |  |
|  | **2-Oxoisocaproate** | 1.00 ± 0.17^b^ | 0.74 ± 0.11^b^ | **1.70 ± 0.30^a^** | **3.07 ± 0.17^a^** | 24.8 | 1.21E-3 | 1.00 ± 0.04 | 0.86 ± 0.04 | 0.82 ± 0.09 | 0.87 ± 0.09 | < 5 | > 0.05 |
|  | **Formate** | 1.00 ± 0.17 | 1.04 ± 0.18 | 0.78 ± 0.07 | 0.76 ± 0.06 | <5 | >0.05 | 1.00 ± 0.12^a^ | **0.56 ± 0.04^b^** | **0.57 ± 0.08^b^** | **0.66 ± 0.01^b^** | 9.83 | 0.0103 |
|  | **Lactate*** | 1.00 ± 0.15^b^ | 0.88 ± 0.07^b^ | 0.81 ± 0.06^b^ | **1.85 ± 0.31^a^** | 10.4 | 9.48E-3 | 1.00 ± 0.13^a^ | 0.95 ± 0.14^a^ | **0.48 ± 0.07^b^** | 0.97 ± 0.13^a^ | 8.15 | 0.0152 |
|  | **2-Phosphoglycerate*** | 1.00 ± 0.02^a^ | 0.84 ± 0.05^a^ | 0.93 ± 0.03^a^ | **0.50 ± 0.02^b^** | 30.5 | 1.21E-3 | 1.00 ± 0.03^bc^ | 1.08 ± 0.06^ab^ | **1.25 ± 0.11^a^** | 0.92 ± 0.08^c^ | 9.87 | 0.0103 |
|  | **O-Phosphoethanolamine** | 1.00 ± 0.06 | 0.80 ± 0.03 | 0.76 ± 0.04 | 0.86 ± 0.06 | < 5 | > 0.05 | 1.00 ± 0.04^a^ | 0.74 ± 0.05^ab^ | **0.66 ± 0.05^b^** | 0.80 ± 0.02^ab^ | 8.21 | 0.0152 |
| **CARBOHYDRATES** | **Fructose** | 1.00 ± 0.05^ab^ | 1.04 ± 0.04^a^ | **0.55 ± 0.04^c^** | 0.87 ± 0.03^b^ | 29.4 | 1.21E-3 | 1.00 ± 0.04^ab^ | 1.11 ± 0.13^a^ | 0.81 ± 0.07^ab^ | **0.72 ± 0.09^b^** | 7.79 | 0.0161 |
|  | **Galactose** | 1.00 ± 0.06^a^ | 0.77 ± 0.01^ab^ | 0.76 ± 0.06^ab^ | **0.63 ± 0.03^b^** | 5.80 | 0.0354 | 1.00 ± 0.06^a^ | 0.69 ± 0.07^ab^ | **0.57 ± 0.05^bc^** | **0.52 ± 0.06^c^** | 15.1 | 4.13E-3 |
|  | **Glucose** | 1.00 ± 0.03 | 0.88 ± 0.04 | 0.77 ± 0.04 | 0.95 ± 0.02 | < 5 | > 0.05 | 1.00 ± 0.15^a^ | 0.68 ± 0.09^ab^ | **0.56 ± 0.06^bc^** | **0.44 ± 0.06^c^** | 10.6 | 8.71E-3 |
|  | **Ribose** | 1.00 ± 0.06 | 1.11 ± 0.06 | 0.99 ± 0.13 | 0.83 ± 0.07 | < 5 | > 0.05 | 1.00 ± 0.08^ab^ | 1.10 ± 0.04^a^ | 0.76 ± 0.05^bc^ | **0.65 ± 0.09^c^** | 13.2 | 5.19E-5 |
|  | **Glycerate** | 1.00 ± 0.08^a^ | 1.02 ± 0.04^a^ | 0.77 ± 0.05^a^ | **0.46 ± 0.03^b^** | 27.6 | 1.21E-3 | 1.00 ± 0.14 | 1.15 ± 0.15 | 0.85 ± 0.11 | 0.94 ± 0.09 | < 5 | > 0.05 |
|  | **Threonate** | nd | nd | nd | nd |  |  | 1.00 ± 0.03 | 0.78 ± 0.06 | 0.73 ± 0.03 | 0.73 ± 0.05 | < 5 | > 0.05 |
|  | **Sucrose** | 1.00 ± 0.08^a^ | 0.97 ± 0.09^a^ | 0.81 ± 0.02^a^ | **0.60 ± 0.04^b^** | 7.83 | 0.0191 | 1.00 ± 0.09 | 0.85 ± 0.05 | 0.99 ± 0.08 | 0.83 ± 0.08 | < 5 | > 0.05 |
|  | **Trehalose** | 1.00 ± 0.06^a^ | 0.95 ± 0.07^a^ | 0.90 ± 0.06^a^ | **0.68 ± 0.01^b^** | 13.6 | 5.08E-3 | 1.00 ± 0.02^a^ | 0.92 ± 0.07^a^ | **0.51 ± 0.01^b^** | **0.62 ± 0.03^b^** | 34.1 | 7.88E-4 |
| **FA** | **2-Hydroxybutyrate** | 1.00 ± 0.15 | 0.83 ± 0.06 | 0.67 ± 0.03 | 0.83 ± 0.06 | < 5 | > 0.05 | 1.00 ± 0.11 | 0.96 ± 0.05 | 0.82 ± 0.06 | 0.83 ± 0.09 | < 5 | > 0.05 |
|  | **Glycolate** | 1.00 ± 0.06^ab^ | 0.76 ± 0.08^b^ | 1.11 ± 0.08^a^ | **0.57 ± 0.04^c^** | 16.3 | 3.05E-3 | 1.00 ± 0.10 | 0.91 ± 0.10 | 0.95 ± 0.08 | 0.72 ± 0.03 | < 5 | > 0.05 |
|  | **Acetate** | 1.00 ± 0.03 | 1.06 ± 0.11 | 1.04 ± 0.11 | 0.89 ± 0.06 | < 5 | > 0.05 | 1.00 ± 0.08 | 0.97 ± 0.07 | 0.83 ± 0.06 | 0.90 ± 0.05 | < 5 | > 0.05 |
|  | **Caprate** | 1.00 ± 0.05 | 0.78 ± 0.03 | 0.65 ± 0.05 | 0.85 ± 0.10 | < 5 | > 0.05 | 1.00 ± 0.02 | 0.86 ± 0.06 | 0.72 ± 0.10 | 1.07 ± 0.09 | < 5 | > 0.05 |
| **ONC** | **Cadaverine** | 1.00 ± 0.15^b^ | 0.99 ± 0.16^b^ | 1.31 ± 0.34^ab^ | **2.46 ± 0.25^a^** | 7.98 | 0.0191 | 1.00 ± 0.05^b^ | 1.41 ± 0.16^b^ | **4.36 ± 0.89^a^** | 2.47 ± 0.54^ab^ | 11.2 | 7.82E-3 |
|  | **Putrescine** | 1.00 ± 0.05^a^ | 0.87 ± 0.09^a^ | 0.65 ± 0.01^ab^ | **0.49 ± 0.06^b^** | 11.4 | 8.08E-3 | 1.00 ± 0.04^a^ | 0.90 ± 0.14^a^ | 0.77 ± 0.07^ab^ | **0.65 ± 0.06^b^** | 6.22 | 0.0274 |
|  | **Ethanolamine** | 1.00 ± 0.06^a^ | **0.72 ± 0.02^b^** | **0.62 ± 0.03^b^** | **0.49 ± 0.04^c^** | 43.0 | 6.61E-4 | 1.00 ± 0.03^a^ | 0.78 ± 0.06^a^ | 0.71 ± 0.06^ab^ | **0.64 ± 0.08^b^** | 7.83 | 0.0161 |
|  | **Choline** | 1.00 ± 0.09^a^ | **0.62 ± 0.04^b^** | **0.55 ± 0.06^b^** | **0.55 ± 0.08^b^** | 12.1 | 6.96E-3 | 1.00 ± 0.09^a^ | 0.77 ± 0.04^a^ | **0.51 ± 0.06^b^** | **0.31 ± 0.04^c^** | 32.6 | 7.88E-4 |
|  | **O-Phosphocholine** | 1.00 ± 0.11^a^ | 0.67 ± 0.09^ab^ | 0.57 ± 0.10^ab^ | **0.51 ± 0.06^b^** | 6.48 | 0.0308 | 1.00 ± 0.03^a^ | 0.76 ± 0.08^a^ | **0.44 ± 0.04^b^** | **0.34 ± 0.05^b^** | 32.8 | 7.88E-4 |
|  | **O-Acetylcholine** | 1.00 ± 0.16^a^ | **0.49 ± 0.05^b^** | 0.53 ± 0.04^ab^ | 0.91 ± 0.12^ab^ | 6.08 | 0.0336 | 1.00 ± 0.06^a^ | 0.74 ± 0.08^a^ | 0.91 ± 0.05^a^ | **0.48 ± 0.05^b^** | 16.1 | 3.75E-3 |
| **OOC** | **Ethanol** | 1.00 ± 0.03 | 1.04 ± 0.11 | 0.99 ± 0.04 | 1.53 ± 0.08 | < 5 | > 0.05 | 1.00 ± 0.12 | 0.72 ± 0.07 | 0.74 ± 0.06 | 1.14 ± 0.07 | < 5 | > 0.05 |
|  | **Glycerol** | 1.00 ± 0.05 | 1.24 ± 0.14 | 1.04 ± 0.10 | 1.70 ± 0.08 | < 5 | > 0.05 | 1.00 ± 0.11 | 0.82 ± 0.04 | 0.63 ± 0.08 | 0.94 ± 0.02 | < 5 | > 0.05 |
|  | **Isopropanol** | 1.00 ± 0.08 | 0.92 ± 0.05 | 0.83 ± 0.06 | 1.00 ± 0.12 | < 5 | > 0.05 | 1.00 ± 0.08 | 0.66 ± 0.06 | 0.70 ± 0.04 | 1.01 ± 0.00 | < 5 | > 0.05 |
| **NUCLEIC ACIDS** | **Adenine** | 1.00 ± 0.13^b^ | 1.16 ± 0.10^ab^ | **1.68 ± 0.21^a^** | 1.40 ± 0.13^ab^ | 8.58 | 0.0155 | 1.00 ± 0.09 | 1.39 ± 0.14 | 1.45 ± 0.13 | 1.52 ± 0.13 | < 5 | > 0.05 |
|  | **Hypoxanthine** | 1.00 ± 0.13 | 0.83 ± 0.08 | 0.76 ± 0.11 | 0.77 ± 0.03 | < 5 | > 0.05 | 1.00 ± 0.03^b^ | **1.58 ± 0.09^a^** | 1.18 ± 0.09^b^ | **0.45 ± 0.04^c^** | 154 | 1.44E-5 |
|  | **Adenosine*** | 1.00 ± 0.03^a^ | 0.94 ± 0.10^a^ | 0.77 ± 0.11^a^ | **0.36 ± 0.02**^b^ | 22.9 | 1.32E-3 | 1.00 ± 0.10^b^ | 1.06 ± 0.07^b^ | **2.76 ± 0.03^a^** | **3.66 ± 0.06^a^** | 86.6 | 6.87E-5 |
|  | **Guanosine** | 1.00 ± 0.14^a^ | 0.79 ± 0.09^ab^ | **0.54 ± 0.06^bc^** | **0.26 ± 0.01^c^** | 17.9 | 2.47E-3 | 1.00 ± 0.14 | 0.89 ± 0.12 | 1.49 ± 0.16 | 1.65 ± 0.17 | 5.15 | 0.0403 |
|  | **Inosine** | 1.00 ± 0.11^a^ | 0.88 ± 0.06^a^ | **0.42 ± 0.03^b^** | **0.36 ± 0.02^c^** | 88.4 | 1.27E-4 | 1.00 ± 0.05^a^ | **0.54 ± 0.03^b^** | 1.33 ± 0.13^a^ | 1.00 ± 0.07^a^ | 18.0 | 3.05E-3 |
|  | **ADP** | 1.00 ± 0.08^c^ | 1.35 ± 0.14^bc^ | **1.38 ± 0.11^b^** | **2.92 ± 0.31^a^** | 24.4 | 1.21E-3 | 1.00 ± 0.12^b^ | 0.85 ± 0.09^b^ | **1.84 ± 0.22^a^** | **2.81 ± 0.11^a^** | 24.1 | 2.06E-3 |
|  | **AMP** | 1.00 ± 0.05^c^ | 1.28 ± 0.09^bc^ | **1.50 ± 0.28^b^** | **2.71 ± 0.11^a^** | 18.4 | 2.37E-3 | 1.00 ± 0.05^b^ | 0.98 ± 0.06^b^ | **1.91 ± 0.24^a^** | **2.75 ± 0.38^a^** | 20.0 | 2.95E-3 |
|  | **ATP^#^** | 1.00 ± 0.07^a^ | 0.67 ± 0.01^ab^ | 0.65 ± 0.02^ab^ | **0.63 ± 0.05^b^** | 6.23 | 0.0332 | 1.00 ± 0.05^b^ | 1.07 ± 0.07^b^ | **1.66 ± 0.18^a^** | **1.74 ± 0.09^a^** | 21.4 | 2.87E-3 |
|  | **GTP** | 1.00 ± 0.03 | 0.91 ± 0.11 | 0.79 ± 0.06 | 0.63 ± 0.07 | < 5 | > 0.05 | 1.00 ± 0.05^c^ | 1.06 ± 0.15^bc^ | **2.05 ± 0.16^a^** | **1.53 ± 0.07^ab^** | 16.3 | 3.75E-3 |
|  | **S-Adenosylhomocysteine** | 1.00 ± 0.11^a^ | 0.93 ± 0.04^a^ | **0.63 ± 0.02^b^** | **0.57 ± 0.04^b^** | 20.3 | 1.79E-3 | 1.00 ± 0.08 | 0.84 ± 0.10 | 0.79 ± 0.04 | 0.90 ± 0.04 | < 5 | > 0.05 |
|  | **Xanthine** | 1.00 ± 0.06^bc^ | 1.28 ± 0.18^ab^ | **1.36 ± 0.11^a^** | 0.77 ± 0.04^c^ | 12.4 | 6.58E-3 | 1.00 ± 0.13^ab^ | 0.69 ± 0.07^b^ | 1.13 ± 0.11^ab^ | **1.31 ± 0.11^a^** | 5.03 | 0.0418 |
|  | **Cytidine** | 1.00 ± 0.05^c^ | **1.57 ± 0.05^a^** | **1.19 ± 0.05^b^** | 1.07 ± 0.03^c^ | 24.8 | 1.21E-3 | nd | nd | nd | nd |  |  |
|  | **Uridine** | 1.00 ± 0.03^a^ | 0.93 ± 0.08^a^ | 0.67 ± 0.07^a^ | **0.35 ± 0.01^b^** | 37.6 | 8.16E-4 | 1.00 ± 0.06^b^ | 0.65 ± 0.10^b^ | **2.24 ± 0.12^a^** | **2.41 ± 0.26^a^** | 34.9 | 7.88E-4 |
|  | **UMP** | 1.00 ± 0.09^b^ | 0.99 ± 0.03^b^ | **1.48 ± 0.08**^a^ | 1.56 ± 0.18^ab^ | 5.90 | 0.0346 | 1.00 ± 0.13^b^ | **1.82 ± 0.28^a^** | **2.27 ± 0.18^a^** | **3.05 ± 0.18^a^** | 17.3 | 3.30E-3 |
|  | **dCTP** | 1.00 ± 0.08^a^ | 0.84 ± 0.03^a^ | **0.64 ± 0.02^b^** | **0.78 ± 0.06^b^** | 25.8 | 1.21E-3 | nd | nd | nd | nd |  |  |
|  | **dTTP** | 1.00 ± 0.09^a^ | **0.70 ± 0.03^b^** | **0.64 ± 0.01^b^** | **0.58 ± 0.04^c^** | 27.0 | 1.21E-3 | 1.00 ± 0.03 | 0.84 ± 0.04 | 0.69 ± 0.06 | 0.98 ± 0.11 | < 5 | > 0.05 |
|  | **Cytosine** | 1.00 ± 0.03^bc^ | 0.99 ± 0.07^b^ | 0.71 ± 0.04^c^ | **1.58 ± 0.02^a^** | 21.3 | 1.60E-3 | 1.00 ± 0.13 | 0.93 ± 0.09 | 1.25 ± 0.16 | 1.64 ± 0.20 | < 5 | > 0.05 |
|  | **Uracil** | 1.00 ± 0.06 | 0.91 ± 0.05 | 0.93 ± 0.13 | 1.15 ± 0.03 | < 5 | > 0.05 | 1.00 ± 0.02^a^ | 0.81 ± 0.05^ab^ | **0.74 ± 0.03^b^** | **0.85 ± 0.06^b^** | 8.29 | 0.0152 |
|  | **Thymine** | 1.00 ± 0.11 | 0.86 ± 0.05 | 0.80 ± 0.07 | 0.91 ± 0.07 | < 5 | > 0.05 | 1.00 ± 0.04^a^ | **0.72 ± 0.01^b^** | **0.65 ± 0.03^b^** | **0.81 ± 0.02^b^** | 13.2 | 5.19E-3 |
| **AK.** | **Niacinamide** | 1.00 ± 0.04^c^ | **1.19 ± 0.05^b^** | **1.44 ± 0.15^ab^** | **1.63 ± 0.06^a^** | 23.9 | 1.21E-3 | 1.00 ± 0.07^b^ | 1.13 ± 0.14^ab^ | **1.74 ± 0.19^a^** | **1.93^a^ ± 0.11** | 10.7 | 8.65E-3 |
|  | **Nicotinate** | 1.00 ± 0.26 | 1.03 ± 0.14 | 0.83 ± 0.09 | 0.62 ± 0.07 | < 5 | > 0.05 | 1.00 ± 0.10^a^ | 0.85 ± 0.09^a^ | **0.42 ± 0.03^b^** | **0.58 ± 0.04^b^** | 18.6 | 3.05E-3 |

* Shapiro-Wilk normality test p <0.05 (green: BW25113, red: Δ*glnP*)

**^#^** Bartlett test of homogeneity of variances p<0.05 (green: BW25113, red: Δ*glnP*)
