# Supplementary material for: Metabolic responses to benzoic acid stress and glutamine transport-dependent vulnerabilities in Escherichia coli revealed by NMR metabolomics
Source: World J Microbiol Biotechnol. 2026 Apr 24;42(5):230. doi: 10.1007/s11274-026-04971-5 (PMC13106250; doi:10.1007/s11274-026-04971-5)
Supplement: Supplementary file 5 — Supplementary Material 5 (DOCX 17.1 KB) [file 11274_2026_4971_MOESM5_ESM.docx]

**­Table S2.** Chemical taxonomy of metabolites detected by ^1^H-NMR in polar extracts of *E. coli* BW25113 and Δ*glnP* cultured in LB medium in the presence and absence of benzoic acid. Metabolite class assignments were based on the Metabolomics Workbench metabolite database (<https://www.metabolomicsworkbench.org/databases/metabolitedatabase.php>).

| **Compound name** | **Super class** | **Main class** | **Sub class** |
| --- | --- | --- | --- |
| 2-Aminobutyrate | Organic acids | Amino acids and peptides | Amino acids |
| Alanine | Organic acids | Amino acids and peptides | Amino acids |
| Arginine | Organic acids | Amino acids and peptides | Amino acids |
| Aspartate | Organic acids | Amino acids and peptides | Amino acids |
| Betaine | Organic acids | Amino acids and peptides | Amino acids |
| Glutamate | Organic acids | Amino acids and peptides | Amino acids |
| Glutamine | Organic acids | Amino acids and peptides | Amino acids |
| Glycine | Organic acids | Amino acids and peptides | Amino acids |
| Histidine | Organic acids | Amino acids and peptides | Amino acids |
| Isoleucine | Organic acids | Amino acids and peptides | Amino acids |
| Leucine | Organic acids | Amino acids and peptides | Amino acids |
| Lysine | Organic acids | Amino acids and peptides | Amino acids |
| Methionine | Organic acids | Amino acids and peptides | Amino acids |
| Pantothenate | Organic acids | Amino acids and peptides | Amino acids |
| Phenylalanine | Organic acids | Amino acids and peptides | Amino acids |
| Proline | Organic acids | Amino acids and peptides | Amino acids |
| Pyroglutamate | Organic acids | Amino acids and peptides | Amino acids |
| Serine | Organic acids | Amino acids and peptides | Amino acids |
| Threonine | Organic acids | Amino acids and peptides | Amino acids |
| Tyrosine | Organic acids | Amino acids and peptides | Amino acids |
| Valine | Organic acids | Amino acids and peptides | Amino acids |
| Glycylproline | Organic acids | Amino acids and peptides | Dipeptides |
| Glutathione | Organic acids | Amino acids and peptides | Tripeptides |
| Fumarate | Organic acids | TCA acids | TCA acids |
| Isocitrate | Organic acids | TCA acids | TCA acids |
| Malate | Organic acids | TCA acids | TCA acids |
| Succinate | Organic acids | TCA acids | TCA acids |
| 2-Oxoisocaproate | Organic acids | Keto acids | Short-chain keto acids |
| Formate | Organic acids | Carboxylic acids | Carboxylic acids |
| Lactate | Organic acids | Short-chain acids | Short-chain acids |
| 2-Phosphoglycerate | Organic acids | Short-chain acids | Short-chain acids |
| O-Phosphoethanolamine | Organic acids | Phosphate esters | Phosphoethanolamines |
| Fructose | Carbohydrates | Monosaccharides | Hexoses |
| Galactose | Carbohydrates | Monosaccharides | Hexoses |
| Glucose | Carbohydrates | Monosaccharides | Hexoses |
| Ribose | Carbohydrates | Monosaccharides | Pentoses |
| Glycerate | Carbohydrates | Monosaccharides | Sugar acids |
| Threonate | Carbohydrates | Monosaccharides | Sugar acids |
| Sucrose | Carbohydrates | Disaccharides | Disaccharides |
| Trehalose | Carbohydrates | Disaccharides | Disaccharides |
| 2-Hydroxybutyrate | Fatty acyls | Fatty acids | Hydroxy FA |
| Glycolate | Fatty acyls | Fatty acids | Hydroxy FA |
| Acetate | Fatty acyls | Fatty acids | Saturated FA |
| Caprate | Fatty acyls | Fatty acids | Saturated FA |
| Cadaverine | Organic nitrogen compounds | Amines | Amines |
| Putrescine | Organic nitrogen compounds | Amines | Amines |
| Ethanolamine | Organic nitrogen compounds | Amines | 1,2-aminoalcohols |
| Choline | Organic nitrogen compounds | Cholines | Cholines |
| O-Phosphocholine | Organic nitrogen compounds | Cholines | Cholines |
| O-Acetylcholine | Organic nitrogen compounds | Cholines | Acyl cholines |
| Ethanol | Organic oxygen compounds | Primary alcohols | Primary alcohols |
| Glycerol | Organic oxygen compounds | Alcohols and polyols | 1,2-diols |
| Isopropanol | Organic oxygen compounds | Alcohols and polyols | Secondary alcohols |
| Adenine | Nucleic acids | Purines | Aminopurines |
| Hypoxanthine | Nucleic acids | Purines | Hypoxanthines |
| Adenosine | Nucleic acids | Purines | Purine ribonucleosides |
| Guanosine | Nucleic acids | Purines | Purine ribonucleosides |
| Inosine | Nucleic acids | Purines | Purine ribonucleosides |
| ADP | Nucleic acids | Purines | Purine rNDP |
| AMP | Nucleic acids | Purines | Purine rNMP |
| ATP | Nucleic acids | Purines | Purine rNTP |
| GTP | Nucleic acids | Purines | Purine dNTP |
| S-Adenosylhomocysteine | Nucleic acids | Purines | Other purines |
| Xanthine | Nucleic acids | Purines | Xanthines |
| Cytidine | Nucleic acids | Pyrimidines | Pyrimidine ribonucleosides |
| Uridine | Nucleic acids | Pyrimidines | Pyrimidine ribonucleosides |
| UMP | Nucleic acids | Pyrimidines | Pyrimidine rNMP |
| dCTP | Nucleic acids | Pyrimidines | Pyrimidine dNTP |
| dTTP | Nucleic acids | Pyrimidines | Pyrimidine dNTP |
| Cytosine | Nucleic acids | Pyrimidines | Pyrimidones |
| Uracil | Nucleic acids | Pyrimidines | Other pyrimidines |
| Thymine | Nucleic acids | Pyrimidines | Other pyrimidines |
| Niacinamide | Alkaloids | Pyridine alkaloids | Nicotinic acid alkaloids |
| Nicotinate | Alkaloids | Pyridine alkaloids | Nicotinic acid alkaloids |
