# Supplementary material for: Metabolic responses to benzoic acid stress and glutamine transport-dependent vulnerabilities in Escherichia coli revealed by NMR metabolomics
Source: World J Microbiol Biotechnol. 2026 Apr 24;42(5):230. doi: 10.1007/s11274-026-04971-5 (PMC13106250; doi:10.1007/s11274-026-04971-5)
Supplement: Supplementary file 6 — Supplementary Material 6 (DOCX 15.3 KB) [file 11274_2026_4971_MOESM6_ESM.docx]

**Table S3.** Quantitative pathway analysis for *E. coli* BW25113 (pink) and Δ*glnP* (green) exposed to 0.25 mg mL^-1^ benzoic acid. Holm-adjusted *p* values were obtained by correcting raw *p* values from enrichment analysis using the Holm-Bonferroni method implemented in the Metaboanalyst 6.0 Pathway Analysis module. Pathways significantly affected by benzoic acid treatment (Holm *p* *<*0.05) are highlighted in gray.

| BW control vs 0.25 mg/mL benzoic acid | Total Cmpd | Hits | Raw p | #AD? | Holm adjust | FDR | Impact |
| --- | --- | --- | --- | --- | --- | --- | --- |
| Glycerophospholipid metabolism | 22 | 2 | 0.000351 | 3.4552 | 0.017882 | 0.012751 | 0.03021 |
| One carbon pool by folate | 20 | 7 | 0.0005 | 3.301 | 0.025003 | 0.012751 | 0.13349 |
| Cyanoamino acid metabolism | 17 | 3 | 0.003953 | 2.4031 | 0.19369 | 0.066077 | 0 |
| Glycine, serine and threonine metabolism | 33 | 7 | 0.005183 | 2.2855 | 0.24876 | 0.066077 | 0.45348 |
| beta-Alanine metabolism | 13 | 3 | 0.007265 | 2.1387 | 0.34147 | 0.074107 | 0 |
| Pantothenate and CoA biosynthesis | 24 | 4 | 0.010673 | 1.9717 | 0.49098 | 0.089665 | 0.14358 |
| Cysteine and methionine metabolism | 42 | 5 | 0.01343 | 1.8719 | 0.60436 | 0.089665 | 0.175 |
| Monobactam biosynthesis | 8 | 1 | 0.014065 | 1.8519 | 0.61887 | 0.089665 | 0 |
| Nicotinate and nicotinamide metabolism | 15 | 4 | 0.046523 | 1.3323 | 1 | 0.26211 | 0.08853 |
| Biosynthesis of various plant secondary metabolites | 6 | 1 | 0.05161 | 1.2873 | 1 | 0.26211 | 0 |
| glnP control vs 0.25 mg/mL benzoic acid | Total Cmpd | Hits | Raw p | #AD? | Holm adjust | FDR | Impact |
| Purine metabolism | 76 | 11 | 0.002273 | 2.6434 | 0.11593 | 0.11593 | 0.15927 |
| Methane metabolism | 26 | 5 | 0.012204 | 1.9135 | 0.61021 | 0.21456 | 0.18399 |
| Pentose phosphate pathway | 26 | 2 | 0.013078 | 1.8835 | 0.64081 | 0.21456 | 0 |
| Pyruvate metabolism | 27 | 6 | 0.020434 | 1.6896 | 0.98083 | 0.21456 | 0.1983 |
| Cyanoamino acid metabolism | 17 | 3 | 0.037818 | 1.4223 | 1 | 0.21456 | 0 |
| beta-Alanine metabolism | 13 | 3 | 0.042008 | 1.3767 | 1 | 0.21456 | 0 |
| Pyrimidine metabolism | 51 | 7 | 0.043258 | 1.3639 | 1 | 0.21456 | 0.298 |
| Histidine metabolism | 12 | 1 | 0.046254 | 1.3349 | 1 | 0.21456 | 0 |
| Monobactam biosynthesis | 8 | 1 | 0.048144 | 1.3175 | 1 | 0.21456 | 0 |
| Other carbon fixation pathways | 24 | 5 | 0.049033 | 1.3095 | 1 | 0.21456 | 0.26421 |
| Biosynthesis of various plant secondary metabolites | 6 | 1 | 0.049342 | 1.3068 | 1 | 0.21456 | 0 |
