# Supplementary material for: Metabolic responses to benzoic acid stress and glutamine transport-dependent vulnerabilities in Escherichia coli revealed by NMR metabolomics
Source: World J Microbiol Biotechnol. 2026 Apr 24;42(5):230. doi: 10.1007/s11274-026-04971-5 (PMC13106250; doi:10.1007/s11274-026-04971-5)
Supplement: Supplementary file 7 — Supplementary Material 7 (DOCX 20.4 KB) [file 11274_2026_4971_MOESM7_ESM.docx]

**Table S5.** Quantitative pathway analysis for *E. coli* BW25113 (pink) and Δ*glnP* (green) exposed to 0.75 mg mL^-1^ benzoic acid. Holm-adjusted *p* values were obtained by correcting raw *p* values from enrichment analysis using the Holm-Bonferroni method implemented in the Metaboanalyst 6.0 Pathway Analysis module. Pathways significantly affected by benzoic acid treatment (Holm *p* *<*0.05) are highlighted in gray.

| BW control vs 0.75 g/mL benzoic acid | Total Cmpd | Hits | Raw p | #AD? | Holm adjust | FDR | Impact |
| --- | --- | --- | --- | --- | --- | --- | --- |
| Purine metabolism | 76 | 11 | 1.16E-05 | 4.934 | 0.000594 | 0.000594 | 0.15927 |
| Glycine, serine and threonine metabolism | 33 | 7 | 4.00E-05 | 4.3974 | 0.002002 | 0.000929 | 0.45348 |
| One carbon pool by folate | 20 | 7 | 5.47E-05 | 4.2624 | 0.002678 | 0.000929 | 0.13349 |
| Glyoxylate and dicarboxylate metabolism | 37 | 10 | 7.57E-05 | 4.1209 | 0.003633 | 0.000965 | 0.18584 |
| Cyanoamino acid metabolism | 17 | 3 | 0.000122 | 3.9142 | 0.005727 | 0.001243 | 0 |
| Glycerolipid metabolism | 15 | 2 | 0.000274 | 3.5616 | 0.012624 | 0.002333 | 0.21905 |
| Lysine degradation | 22 | 3 | 0.000535 | 3.2716 | 0.024076 | 0.003898 | 0.1267 |
| Lysine biosynthesis | 13 | 2 | 0.000745 | 3.1279 | 0.032779 | 0.004699 | 0 |
| Glycerophospholipid metabolism | 22 | 2 | 0.000829 | 3.0813 | 0.035658 | 0.004699 | 0.03021 |
| Cysteine and methionine metabolism | 42 | 5 | 0.000926 | 3.0336 | 0.038877 | 0.004721 | 0.175 |
| Nicotinate and nicotinamide metabolism | 15 | 4 | 0.001092 | 2.962 | 0.044752 | 0.005061 | 0.08853 |
| D-Amino acid metabolism | 25 | 6 | 0.001854 | 2.732 | 0.07415 | 0.007878 | 0.08696 |
| Lipoic acid metabolism | 28 | 1 | 0.002882 | 2.5403 | 0.1124 | 0.011306 | 0.00176 |
| Methane metabolism | 26 | 4 | 0.003339 | 2.4763 | 0.1269 | 0.012165 | 0.18399 |
| Pyrimidine metabolism | 51 | 9 | 0.005282 | 2.2772 | 0.19543 | 0.01728 | 0.33374 |
| Glutathione metabolism | 22 | 6 | 0.005421 | 2.2659 | 0.19543 | 0.01728 | 0.47191 |
| Galactose metabolism | 39 | 5 | 0.007574 | 2.1207 | 0.2651 | 0.022722 | 0.33019 |
| beta-Alanine metabolism | 13 | 3 | 0.009788 | 2.0093 | 0.33277 | 0.027731 | 0 |
| Pyruvate metabolism | 27 | 5 | 0.011688 | 1.9322 | 0.38572 | 0.031374 | 0.06669 |
| Valine, leucine and isoleucine biosynthesis | 22 | 5 | 0.015631 | 1.806 | 0.50019 | 0.039859 | 0 |
| Starch and sucrose metabolism | 22 | 4 | 0.017498 | 1.757 | 0.54242 | 0.040649 | 0.31509 |
| Pantothenate and CoA biosynthesis | 24 | 4 | 0.017535 | 1.7561 | 0.54242 | 0.040649 | 0.14358 |
| Arginine biosynthesis | 16 | 4 | 0.019744 | 1.7046 | 0.57257 | 0.043779 | 0.1044 |
| Valine, leucine and isoleucine degradation | 22 | 4 | 0.023354 | 1.6316 | 0.65392 | 0.049628 | 0 |
| Phenylalanine, tyrosine and tryptophan biosynthesis | 23 | 2 | 0.026723 | 1.5731 | 0.72152 | 0.054515 | 0.00046 |
| Monobactam biosynthesis | 8 | 1 | 0.028316 | 1.548 | 0.73621 | 0.055542 | 0 |
| Glycolysis or Gluconeogenesis | 29 | 3 | 0.03404 | 1.468 | 0.85099 | 0.064297 | 0.0239 |
| Folate biosynthesis | 38 | 1 | 0.036742 | 1.4348 | 0.88181 | 0.064615 | 0.06924 |
| Riboflavin metabolism | 17 | 1 | 0.036742 | 1.4348 | 0.88181 | 0.064615 | 0 |
| Alanine, aspartate and glutamate metabolism | 22 | 5 | 0.041942 | 1.3774 | 0.92272 | 0.071301 | 0.65769 |
| Phenylalanine metabolism | 35 | 2 | 0.04616 | 1.3357 | 0.96937 | 0.075941 | 0.00347 |
| glnP control vs 0.75 g/mL benzoic acid | Total Cmpd | Hits | Raw p | #AD? | Holm adjust | FDR | Impact |
| Arginine biosynthesis | 16 | 5 | 0.000133 | 3.8758 | 0.006788 | 0.004033 | 0.1044 |
| Other carbon fixation pathways | 24 | 5 | 0.000167 | 3.7778 | 0.008341 | 0.004033 | 0.26421 |
| D-Amino acid metabolism | 25 | 6 | 0.000351 | 3.4543 | 0.017213 | 0.004033 | 0.08696 |
| Glyoxylate and dicarboxylate metabolism | 37 | 10 | 0.000361 | 3.442 | 0.017346 | 0.004033 | 0.27843 |
| One carbon pool by folate | 20 | 7 | 0.000446 | 3.3504 | 0.020977 | 0.004033 | 0.13349 |
| Lysine degradation | 22 | 2 | 0.000474 | 3.3238 | 0.021824 | 0.004033 | 0.1267 |
| Cyanoamino acid metabolism | 17 | 3 | 0.00056 | 3.2517 | 0.025208 | 0.004081 | 0 |
| Methane metabolism | 26 | 5 | 0.000708 | 3.1499 | 0.031157 | 0.004186 | 0.18399 |
| Cysteine and methionine metabolism | 42 | 5 | 0.000739 | 3.1315 | 0.031765 | 0.004186 | 0.175 |
| Pyrimidine metabolism | 51 | 7 | 0.00115 | 2.9392 | 0.048306 | 0.005866 | 0.298 |
| Arginine and proline metabolism | 29 | 4 | 0.001336 | 2.8741 | 0.054785 | 0.006195 | 0.33989 |
| Purine metabolism | 76 | 11 | 0.001475 | 2.8313 | 0.058988 | 0.006268 | 0.15927 |
| Phenylalanine metabolism | 35 | 2 | 0.00202 | 2.6946 | 0.078792 | 0.007439 | 0.00347 |
| Glutathione metabolism | 22 | 6 | 0.002042 | 2.69 | 0.078792 | 0.007439 | 0.47191 |
| Glycerophospholipid metabolism | 22 | 2 | 0.002644 | 2.5778 | 0.097813 | 0.008988 | 0.03021 |
| Nitrogen metabolism | 11 | 2 | 0.004397 | 2.3569 | 0.15827 | 0.014014 | 0 |
| Nicotinate and nicotinamide metabolism | 15 | 3 | 0.005154 | 2.2878 | 0.1804 | 0.0154 | 0.08853 |
| Alanine, aspartate and glutamate metabolism | 22 | 5 | 0.005435 | 2.2648 | 0.1848 | 0.0154 | 0.66154 |
| Streptomycin biosynthesis | 9 | 1 | 0.007109 | 2.1482 | 0.23458 | 0.019081 | 0 |
| Lysine biosynthesis | 13 | 2 | 0.007839 | 2.1058 | 0.25083 | 0.019988 | 0 |
| Monobactam biosynthesis | 8 | 1 | 0.008546 | 2.0683 | 0.26491 | 0.020754 | 0 |
| Folate biosynthesis | 38 | 1 | 0.009698 | 2.0133 | 0.29095 | 0.021505 | 0.06924 |
| Riboflavin metabolism | 17 | 1 | 0.009698 | 2.0133 | 0.29095 | 0.021505 | 0 |
| Amino sugar and nucleotide sugar metabolism | 44 | 2 | 0.010552 | 1.9767 | 0.29545 | 0.022191 | 0 |
| Glycine, serine and threonine metabolism | 33 | 7 | 0.010878 | 1.9634 | 0.29545 | 0.022191 | 0.45348 |
| Starch and sucrose metabolism | 22 | 4 | 0.01206 | 1.9187 | 0.31355 | 0.023655 | 0.31509 |
| Galactose metabolism | 39 | 5 | 0.012979 | 1.8868 | 0.32448 | 0.024295 | 0.33019 |
| Carbon fixation by Calvin cycle | 18 | 1 | 0.013421 | 1.8722 | 0.32448 | 0.024295 | 0.0283 |
| Pentose phosphate pathway | 26 | 2 | 0.013815 | 1.8597 | 0.32448 | 0.024295 | 0 |
| Citrate cycle (TCA cycle) | 20 | 3 | 0.016193 | 1.7907 | 0.35624 | 0.027528 | 0.09959 |
| Pyruvate metabolism | 27 | 6 | 0.017115 | 1.7666 | 0.35942 | 0.028157 | 0.1983 |
| Pantothenate and CoA biosynthesis | 24 | 4 | 0.019069 | 1.7197 | 0.38139 | 0.02993 | 0.14358 |
| Sulfur metabolism | 20 | 2 | 0.019366 | 1.713 | 0.38139 | 0.02993 | 0.04281 |
| Lipoic acid metabolism | 28 | 1 | 0.021426 | 1.6691 | 0.38566 | 0.032138 | 0.00176 |
| beta-Alanine metabolism | 13 | 3 | 0.025195 | 1.5987 | 0.42832 | 0.036713 | 0 |
| Fructose and mannose metabolism | 33 | 1 | 0.034241 | 1.4655 | 0.54786 | 0.048508 | 0.05259 |
| Biosynthesis of various plant secondary metabolites | 6 | 1 | 0.045081 | 1.346 | 0.67621 | 0.059566 | 0 |
| Phenylalanine, tyrosine and tryptophan biosynthesis | 23 | 2 | 0.046618 | 1.3314 | 0.67621 | 0.059566 | 0.00046 |
| Valine, leucine and isoleucine degradation | 22 | 4 | 0.046715 | 1.3305 | 0.67621 | 0.059566 | 0 |
| Histidine metabolism | 12 | 1 | 0.047699 | 1.3215 | 0.67621 | 0.059566 | 0 |
