# Supplementary material for: Metabolic responses to benzoic acid stress and glutamine transport-dependent vulnerabilities in Escherichia coli revealed by NMR metabolomics
Source: World J Microbiol Biotechnol. 2026 Apr 24;42(5):230. doi: 10.1007/s11274-026-04971-5 (PMC13106250; doi:10.1007/s11274-026-04971-5)
Supplement: Supplementary file 8 — Supplementary Material 8 (DOCX 19.3 KB) [file 11274_2026_4971_MOESM8_ESM.docx]

**Table S4.** Quantitative pathway analysis for *E. coli* BW25113 (pink) and Δ*glnP* (green) exposed to 0.50 mg mL^-1^ benzoic acid. Holm-adjusted *p* values were obtained by correcting raw *p* values from enrichment analysis using the Holm-Bonferroni method implemented in the Metaboanalyst 6.0 Pathway Analysis module. Pathways significantly affected by benzoic acid treatment (Holm *p* *<*0.05) are highlighted in gray.

| BW control vs 0.5 mg/mL benzoic acid | Total Cmpd | Hits | Raw p | #AD? | Holm adjust | FDR | Impact |
| --- | --- | --- | --- | --- | --- | --- | --- |
| Pyrimidine metabolism | 51 | 9 | 0.000589 | 3.2297 | 0.030051 | 0.010214 | 0.33374 |
| Purine metabolism | 76 | 11 | 0.000702 | 3.1538 | 0.035087 | 0.010214 | 0.15927 |
| One carbon pool by folate | 20 | 7 | 0.000739 | 3.1312 | 0.036223 | 0.010214 | 0.13349 |
| Lysine biosynthesis | 13 | 2 | 0.000801 | 3.0963 | 0.038454 | 0.010214 | 0 |
| Glycine, serine and threonine metabolism | 33 | 7 | 0.001684 | 2.7736 | 0.079161 | 0.01718 | 0.45348 |
| Arginine biosynthesis | 16 | 4 | 0.004885 | 2.3112 | 0.2247 | 0.030605 | 0.1044 |
| Cyanoamino acid metabolism | 17 | 3 | 0.005224 | 2.282 | 0.23508 | 0.030605 | 0 |
| Fructose and mannose metabolism | 33 | 1 | 0.005869 | 2.2315 | 0.25822 | 0.030605 | 0.05259 |
| Glycerophospholipid metabolism | 22 | 2 | 0.005999 | 2.2219 | 0.25822 | 0.030605 | 0.03021 |
| D-Amino acid metabolism | 25 | 6 | 0.006001 | 2.2218 | 0.25822 | 0.030605 | 0.08696 |
| Glutathione metabolism | 22 | 6 | 0.008867 | 2.0522 | 0.36353 | 0.041108 | 0.47191 |
| Arginine and proline metabolism | 29 | 4 | 0.011823 | 1.9273 | 0.47294 | 0.048651 | 0.33989 |
| Nicotinate and nicotinamide metabolism | 15 | 4 | 0.012401 | 1.9065 | 0.48365 | 0.048651 | 0.08853 |
| Cysteine and methionine metabolism | 42 | 5 | 0.018168 | 1.7407 | 0.69038 | 0.06489 | 0.175 |
| Glyoxylate and dicarboxylate metabolism | 37 | 10 | 0.019085 | 1.7193 | 0.70615 | 0.06489 | 0.18584 |
| beta-Alanine metabolism | 13 | 3 | 0.021178 | 1.6741 | 0.7624 | 0.067504 | 0 |
| Glycolysis or Gluconeogenesis | 29 | 3 | 0.025139 | 1.5997 | 0.87987 | 0.075417 | 0.0239 |
| Amino sugar and nucleotide sugar metabolism | 44 | 2 | 0.030029 | 1.5225 | 1 | 0.080683 | 0 |
| Monobactam biosynthesis | 8 | 1 | 0.030059 | 1.522 | 1 | 0.080683 | 0 |
| Lipoic acid metabolism | 28 | 1 | 0.043093 | 1.3656 | 1 | 0.10989 | 0.00176 |
| Methane metabolism | 26 | 4 | 0.047606 | 1.3223 | 1 | 0.11561 | 0.18399 |
| glnP control vs 0.50 mg/mL benzoic acid | Total Cmpd | Hits | Raw p | #AD? | Holm adjust | FDR | Impact |
| Pyrimidine metabolism | 51 | 7 | 3.06E-05 | 4.5149 | 0.001558 | 0.00083 | 0.298 |
| One carbon pool by folate | 20 | 7 | 3.25E-05 | 4.4875 | 0.001627 | 0.00083 | 0.13349 |
| Folate biosynthesis | 38 | 1 | 0.000188 | 3.7248 | 0.009234 | 0.00195 | 0.06924 |
| Riboflavin metabolism | 17 | 1 | 0.000188 | 3.7248 | 0.009234 | 0.00195 | 0 |
| Glutathione metabolism | 22 | 6 | 0.000191 | 3.7186 | 0.009234 | 0.00195 | 0.47191 |
| beta-Alanine metabolism | 13 | 3 | 0.000676 | 3.1701 | 0.03109 | 0.005745 | 0 |
| Phenylalanine metabolism | 35 | 2 | 0.001037 | 2.984 | 0.046684 | 0.007558 | 0.00347 |
| Lysine degradation | 22 | 2 | 0.001666 | 2.7783 | 0.073307 | 0.010621 | 0.1267 |
| Other carbon fixation pathways | 24 | 5 | 0.002298 | 2.6387 | 0.098812 | 0.012921 | 0.26421 |
| Purine metabolism | 76 | 11 | 0.002868 | 2.5424 | 0.12045 | 0.012921 | 0.15927 |
| Pantothenate and CoA biosynthesis | 24 | 4 | 0.003202 | 2.4947 | 0.13126 | 0.012921 | 0.14358 |
| Cysteine and methionine metabolism | 42 | 5 | 0.003228 | 2.491 | 0.13126 | 0.012921 | 0.175 |
| Pyruvate metabolism | 27 | 6 | 0.003294 | 2.4823 | 0.13126 | 0.012921 | 0.1983 |
| Methane metabolism | 26 | 5 | 0.005222 | 2.2821 | 0.19845 | 0.017563 | 0.18399 |
| Glyoxylate and dicarboxylate metabolism | 37 | 10 | 0.005707 | 2.2436 | 0.21115 | 0.017563 | 0.27843 |
| Lipoic acid metabolism | 28 | 1 | 0.005802 | 2.2364 | 0.21115 | 0.017563 | 0.00176 |
| Galactose metabolism | 39 | 5 | 0.005854 | 2.2325 | 0.21115 | 0.017563 | 0.33019 |
| Glycerophospholipid metabolism | 22 | 2 | 0.006518 | 2.1859 | 0.22162 | 0.018141 | 0.03021 |
| Nicotinate and nicotinamide metabolism | 15 | 3 | 0.006774 | 2.1692 | 0.22353 | 0.018141 | 0.08853 |
| Starch and sucrose metabolism | 22 | 4 | 0.007255 | 2.1394 | 0.23216 | 0.018141 | 0.31509 |
| Glycolysis or Gluconeogenesis | 29 | 3 | 0.00747 | 2.1267 | 0.23216 | 0.018141 | 0.0239 |
| Histidine metabolism | 12 | 1 | 0.008087 | 2.0922 | 0.24259 | 0.018377 | 0 |
| Cyanoamino acid metabolism | 17 | 3 | 0.008288 | 2.0816 | 0.24259 | 0.018377 | 0 |
| Citrate cycle (TCA cycle) | 20 | 3 | 0.009346 | 2.0294 | 0.26168 | 0.01986 | 0.09959 |
| Butanoate metabolism | 18 | 2 | 0.013474 | 1.8705 | 0.36379 | 0.02656 | 0 |
| Arginine biosynthesis | 16 | 5 | 0.013541 | 1.8684 | 0.36379 | 0.02656 | 0.1044 |
| Sulfur metabolism | 20 | 2 | 0.015526 | 1.809 | 0.38814 | 0.029062 | 0.04281 |
| Arginine and proline metabolism | 29 | 4 | 0.015956 | 1.7971 | 0.38814 | 0.029062 | 0.33989 |
| D-Amino acid metabolism | 25 | 6 | 0.016627 | 1.7792 | 0.38814 | 0.029241 | 0.08696 |
| Streptomycin biosynthesis | 9 | 1 | 0.017571 | 1.7552 | 0.38814 | 0.029871 | 0 |
| Amino sugar and nucleotide sugar metabolism | 44 | 2 | 0.018475 | 1.7334 | 0.38814 | 0.030394 | 0 |
| Pentose phosphate pathway | 26 | 2 | 0.021628 | 1.665 | 0.43256 | 0.03447 | 0 |
| Alanine, aspartate and glutamate metabolism | 22 | 5 | 0.023383 | 1.6311 | 0.44428 | 0.035419 | 0.66154 |
| Lysine biosynthesis | 13 | 2 | 0.023613 | 1.6269 | 0.44428 | 0.035419 | 0 |
| Glycine, serine and threonine metabolism | 33 | 7 | 0.028011 | 1.5527 | 0.47619 | 0.040816 | 0.45348 |
| Valine, leucine and isoleucine degradation | 22 | 4 | 0.035238 | 1.453 | 0.5638 | 0.04992 | 0 |
| Nitrogen metabolism | 11 | 2 | 0.037972 | 1.4205 | 0.56959 | 0.05234 | 0 |
| Valine, leucine and isoleucine biosynthesis | 22 | 5 | 0.046903 | 1.3288 | 0.65664 | 0.061611 | 0 |
| Phenylalanine, tyrosine and tryptophan biosynthesis | 23 | 2 | 0.047114 | 1.3269 | 0.65664 | 0.061611 | 0.00046 |
