# Supplementary material for: Metabolic responses to benzoic acid stress and glutamine transport-dependent vulnerabilities in Escherichia coli revealed by NMR metabolomics
Source: World J Microbiol Biotechnol. 2026 Apr 24;42(5):230. doi: 10.1007/s11274-026-04971-5 (PMC13106250; doi:10.1007/s11274-026-04971-5)
Supplement: Supplementary file 9 — Supplementary Material 9 (DOCX 1.13 MB) [file 11274_2026_4971_MOESM9_ESM.docx]

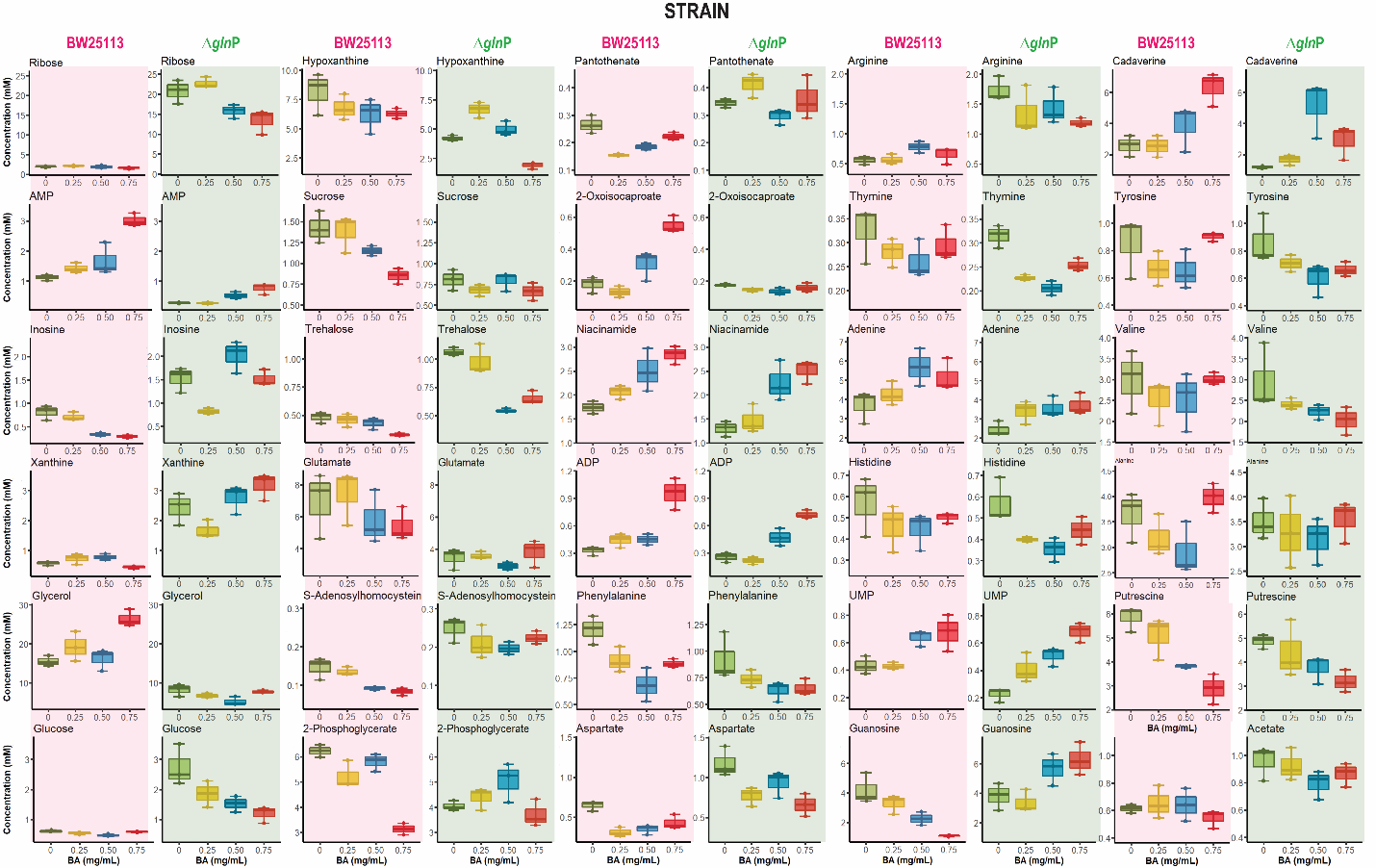


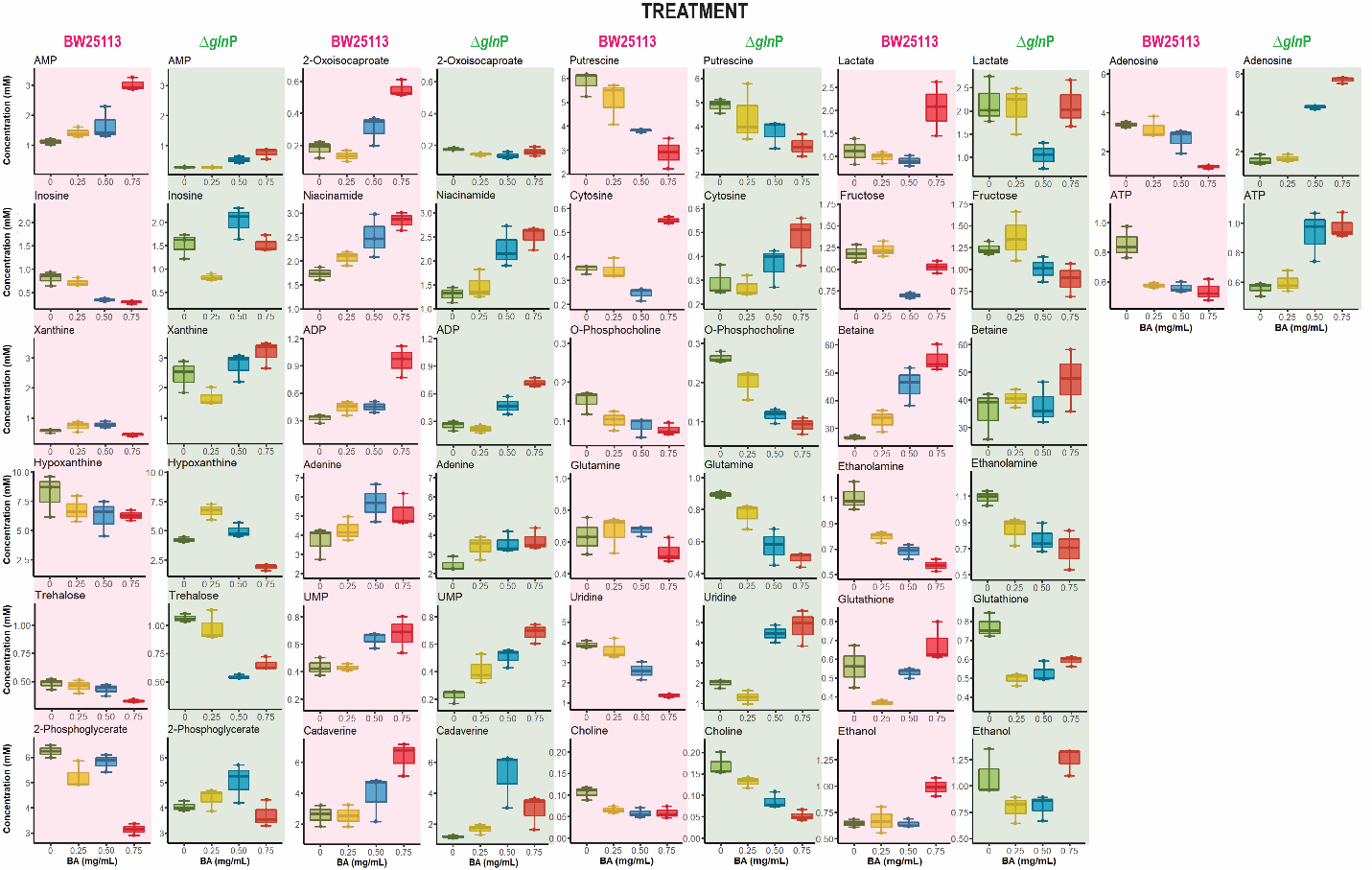


­
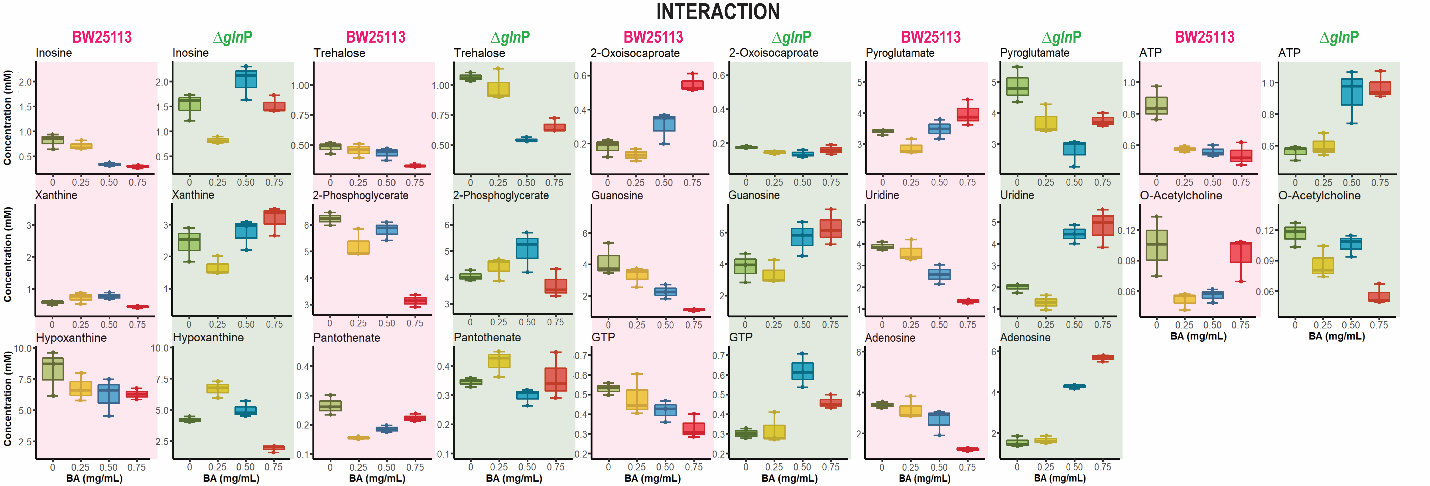


**Figure S3:** **Box plots showing strain- and treatment-dependent changes in metabolite concentrations of** E. coli **BW25113 and ΔglnP cells exposed to benzoic acid.** Metabolites that displayed significant effects of strain (**A**), treatment (**B**), or strain × treatment interactions (**C**) according to two-way ANOVA with Bonferroni-adjusted p < 0.05 are shown. Each panel compares metabolite levels between wild-type BW25113 (pink background) and ΔglnP mutant (green background) under control (0), low (0.25 mg/mL), medium (0.50 mg/mL), and high (0.75 mg/mL) benzoic acid conditions. Metabolite intensities were normalized using total spectral area as scaling factor. Data are represented as box plots for n = 3 biological replicates, showing the median (horizontal line), interquartile range (box; middle 50% of values), and full data range (whiskers).
